# Basal autophagy prevents autoactivation or enhancement of inflammatory signals by targeting monomeric MyD88

**DOI:** 10.1038/s41598-017-01246-w

**Published:** 2017-04-21

**Authors:** Takeshi Into, Toshi Horie, Megumi Inomata, Jin Gohda, Jun-ichiro Inoue, Yukitaka Murakami, Shumpei Niida

**Affiliations:** 1 Department of Oral Microbiology, Division of Oral Infections and Health Sciences, Asahi University School of Dentistry, Mizuho, Japan; 2 Research Center for Asian Infectious Diseases, The Institute of Medical Science, The University of Tokyo, Tokyo, Japan; 3 Division of Cellular and Molecular Biology, The Institute of Medical Science, The University of Tokyo, Tokyo, Japan; 4 Medical Genome Center, National Center for Geriatrics and Gerontology, Obu, Japan

## Abstract

Autophagy, the processes of delivery of intracellular components to lysosomes, regulates induction of inflammation. Inducible macroautophagy degrades inflammasomes and dysfunctional mitochondria to downregulate inflammatory signals. Nonetheless, the effects of constitutive basal autophagy on inflammatory signals are largely unknown. Here, we report a previously unknown effect of basal autophagy. Lysosomal inhibition induced weak inflammatory signals in the absence of a cellular stimulus and in the presence of a nutrient supply, and their induction was impaired by MyD88 deficiency. During lysosomal inhibition, MyD88 was accumulated, and overabundant MyD88 autoactivated downstream signaling or enhanced TLR/IL-1R-mediated signaling. MyD88 is probably degraded via basal microautophagy because macroautophagy inhibitors, ATG5 deficiency, and an activator of chaperone-mediated autophagy did not affect MyD88. Analysis using a chimeric protein whose monomerization/dimerization can be switched revealed that monomeric MyD88 is susceptible to degradation. Immunoprecipitation of monomeric MyD88 revealed its interaction with TRAF6. In TRAF6-deficient cells, degradation of basal MyD88 was enhanced, suggesting that TRAF6 participates in protection from basal autophagy. Thus, basal autophagy lowers monomeric MyD88 expression, and thereby autoactivation of inflammatory signals is prevented. Given that impairment of lysosomes occurs in various settings, our results provide novel insights into the etiology of inflammatory signals that affect consequences of inflammation.

## Introduction

Inflammation is an immunovascular response of tissues to a wide range of stimuli, including microbial pathogens and endogenously generated molecules, where innate immune recognition plays key roles^1, 2^. Pattern recognition receptors (PRRs), including Toll-like receptors (TLRs) and NOD-like receptors (NLRs), can activate primary inflammatory signals after recognition of microbial pathogen-associated molecular patterns (PAMPs) or endogenously generated damage-associated molecular patterns (DAMPs)^1, 2^. TLRs induce a homotypic interaction with an adaptor protein termed myeloid differentiation primary response 88 (MyD88) and initiate intracellular signaling via activation of the E3 ubiquitin ligase tumor necrosis factor receptor-associated factor 6 (TRAF6)^3^. The enzymatic and scaffolding properties of TRAF6 mediate induction of proinflammatory signaling for activation of nuclear factor (NF)-κB and NF-κB-dependent production of proinflammatory cytokines, including tumor necrosis factor (TNF)-α and the precursor of interleukin (IL)-1β (pro-IL-1β)^3, 4^. Members of the NLR family, including NLRP1 and NLRP3, can be activated by PAMPs, DAMPs, and other stimuli, including extracellular ATP and reactive oxygen species (ROS), to initiate assembly of inflammasomes for caspase-1 activation, subsequent caspase-1-mediated cleavage of pro-IL-1β, and a release of mature IL-1β as a key mediator of inflammation^5^. Much progress has been made in the understanding how innate-immunity-associated mechanisms create inflammatory conditions, which affect not only physiological responses but also the development of diseases accompanied by inflammation^1, 2^.

Autophagy, cellular processes characterized by the delivery of intracellular components to lysosomes for degradation, is currently known to counteract inflammatory responses^6, 7^. Basically, autophagy is induced in response to cellular stimuli or nutrient depletion, and its degradative effects through specific or non-specific mechanisms remove unnecessary cytoplasmic proteins and organelles for cellular quality control and thus supply essential nutrients for metabolic control. Autophagy can be induced along with inflammatory signals and eliminates the source of inflammatory stimuli, such as intracellular pathogens and damaged mitochondria, inflammasomes, and signaling molecules downstream of PRRs^8, 9^. Autophagy thus yields multiple effects leading to downregulation of excessive and sustained inflammatory responses. Additionally, many lines of evidence have indicated that defects of autophagy because of genetic factors cause inflammation in autoinflammatory and autoimmune diseases^7, 10^. Failure of autophagy causes accumulation of damaged mitochondria and increases production of ROS, resulting in activation or enhancement of inflammatory signals accompanied by inflammasome activation^9, 11^.

The autophagy–lysosome pathways involve at least three mechanisms: macroautophagy, chaperone-mediated autophagy (CMA), and microautophagy^12^. Macroautophagy corresponds to the de novo formation of double-membrane vesicles termed autophagosomes around a cargo (such as unnecessary or sequestered structures) in order to randomly or specifically degrade it via fusion with lysosomes. The macroautophagy pathway involves the functions of many autophagy-related proteins (ATGs), and the core machinery of autophagosome formation is driven by essential ATGs^13^. CMA is characterized by lysosomal targeting through translocation of target cytosolic proteins (that contain a KFERQ-like pentapeptide motif) across the lysosomal membrane with subsequent degradation^14^. CMA does not require the de novo vesicle formation and is achieved through binding of the targets to the heat shock protein Hsc70/Hspa8, via their transportation to the complex of lysosome-associated membrane protein 2A (LAMP2A) and Hsp90 in the lysosomal membrane, and by bringing the targets to the lysosomal lumen. Microautophagy is a process by which cytoplasmic materials are delivered to the lysosomal lumen via random invaginations of the lysosomal or late endosomal membrane. Autophagy and inflammation are regarded as interdependent processes; however, most of the reported suppressive effects on inflammation are associated with macroautophagy, and the implication of other types of autophagy is unclear. Additionally, although autophagy induction has different dynamics, i.e. cellular stimulus- or nutrient depletion-activated ‘inducible autophagy’ and constitutively active ‘basal autophagy’^15, 16^, distinct effects of basal autophagy on inflammation have not been clarified.

In recent studies, the detailed roles of autophagy–lysosome pathways in the suppression of inflammation have aroused increasing interest. We aimed to test whether basal autophagy, which is constitutively active (even under conditions of abundant nutrients and in the absence of any cellular stimuli) performs such a function. Here, we report that pharmacological inhibition of lysosomes triggers activation of weak inflammatory signals in macrophages. Of note, they are impaired by MyD88 deficiency and yield intracellular accumulation of MyD88. Moreover, accumulated MyD88 during lysosomal inhibition enhances TLR- or IL-1 receptor (IL-1R)-activated inflammatory signals. Conversely, these responses are intrinsically prevented via basal autophagic degradation of MyD88. Our findings highlight a previously unknown preventive effect of basal autophagy on inflammatory signals and imply the involvement of basal authophagy in the regulation of inflammatory processes.

## Results

### Inhibition of lysosomes activates weak inflammatory signals

**Figure 1:**
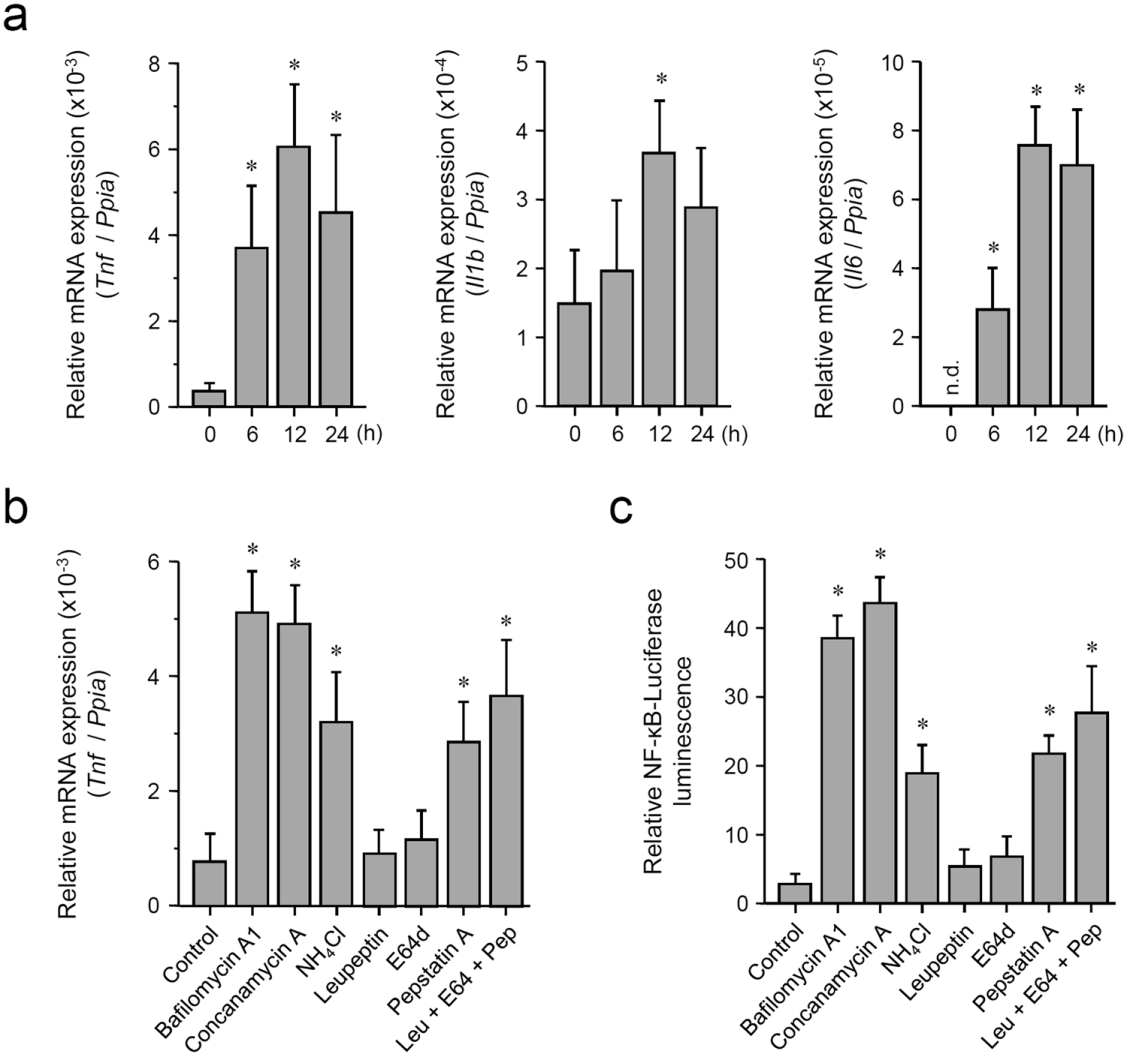
Pharmacological inhibition of lysosomes induces weak inflammatory signals in macrophages. (**a**) Induction of mRNA of inflammatory cytokines by BafA1 treatment. Mouse BMDMs were incubated with 100 nM BafA1 for the indicated periods, followed by RNA extraction. Relative expression levels of *Tnf*, *Il1b*, and *Il6* were determined by qRT-PCR. Each value is expressed as the mean ± SD (n = 3); *p < 0.05 (versus 0 h), one-way ANOVA and Dunnett’s test for post-hoc comparisons (μc < μi). n.d.: not detected (regarded as 0). (**b**) Treatment with the inhibitors of lysosomal functions or lysosomal proteases induces *Tnf* expression. Mouse BMDMs were incubated with 0.5% DMSO (control), 100 nM BafA1, 100 nM concanamycin A, 20 mM ammonium chloride, 50 μM leupeptin, 50 μM E-64-d, or 2 μM pepstatin A for 12 h, followed by RNA extraction. Relative expression levels of *Tnf* were determined by qRT-PCR. Each value is expressed as mean ± SD (n = 3); *p < 0.05 (versus Control), one-way ANOVA and Dunnett’s test for post-hoc comparisons (μc < μi). (**c**) Treatment with the inhibitors of lysosomal functions or lysosomal proteases activates NF-κB-dependent transcriptional activity. The NF-κB-driven luciferase reporter assay was performed in RAW264.7 cells. Cells were incubated with 0.5% DMSO (control), 100 nM BafA1, 100 nM concanamycin A, 20 mM ammonium chloride, 50 μM leupeptin, 50 μM E-64-d, or 2 μM pepstatin A for 12 h. Relative NF-κB activity was measured, and the data are expressed as mean ± SD (n = 3); *p < 0.05 (versus Control), one-way ANOVA and Dunnett’s test for post-hoc comparisons (μc < μi).

To determine whether basal autophagy affects inflammatory signals, we manipulated lysosomes because different mechanisms or pathways of autophagy ultimately lead to lysosomal degradation. Especially, we tested whether treatment of macrophages with a lysosomotropic inhibitor activates inflammatory signals. Bone marrow-derived macrophages (BMDMs) from C57BL/6 (B6) mice were simply incubated with the vacuolar H^+^-ATPase inhibitor bafilomycin A_1_ (BafA1) in the absence of any extracellular stimuli and under normal culture conditions, followed by measurement of mRNA expression of proinflammatory cytokines. As a result, considerably weak but significant induction of mRNA expression of *Tnf*, *Il1b*, and *Il6* was observed after BafA1 treatment (Fig. 1a and Supplementary Fig. 1). The response to BafA1 treatment was also observed in mouse embryonic fibroblasts (MEFs) despite a weaker response than that in BMDMs (Supplementary Fig. 2). Expression of *Tnf* was also induced by concanamycin A, another lysosomal vacuolar-type H^+^-ATPase inhibitor, and by ammonium chloride that accumulates inside lysosomes and neutralizes their intrinsic acidic pH for inhibition of lysosomal protease activities^17^ (Fig. 1b). Treatment with the specific inhibitors of each lysosomal protease was also tested. Lysosomal cysteine protease inhibitor E-64-d and lysosomal serine and cysteine protease inhibitor leupeptin minimally affected *Tnf* expression, while the lysosomal aspartic protease inhibitor pepstatin A clearly induced *Tnf* expression (Fig. 1b). The mixed treatment with these inhibitors resulted in a somewhat larger response as compared to the results of pepstatin A treatment alone (Fig. 1b).

We then tested whether inhibition of the lysosomal function stimulates activation of NF-κB, the most important transcription factor in proinflammatory signaling. The transcriptional activity was assessed by an NF-κB-driven luciferase reporter gene assay in macrophage-like RAW264.7 cells. As in the results on *Tnf* expression, NF-κB activation was observed during treatment with BafA1, concanamycin A, ammonium chloride, or pepstatin A (Fig. 1c). Moreover, treatment with BafA1 or pepstatin A also activated transcriptional activity of AP-1 as assessed by an AP-1-driven reporter gene assay (Supplementary Fig. 3). These results suggest that impairment of lysosomes, especially lysosomal aspartic proteases, leads to activation of inflammatory signals, which may be intrinsically prevented by basal autophagy.

### MyD88 is accumulated and mediates inflammatory signaling during lysosomal impairment

MyD88, the best-known adaptor protein for signal transduction of TLRs and IL-1R, is involved in triggering of the proinflammatory pathway of NF-κB and in activation of AP-1 as a consequence of mitogen-activated protein kinase cascades^3^. Overexpression of MyD88 by gene transfection is known to autoactivate downstream signaling even in the absence of receptor ligation^18^. We therefore tested whether MyD88 is involved in the induction of inflammatory signals during lysosomal inhibition.

**Figure 2:**
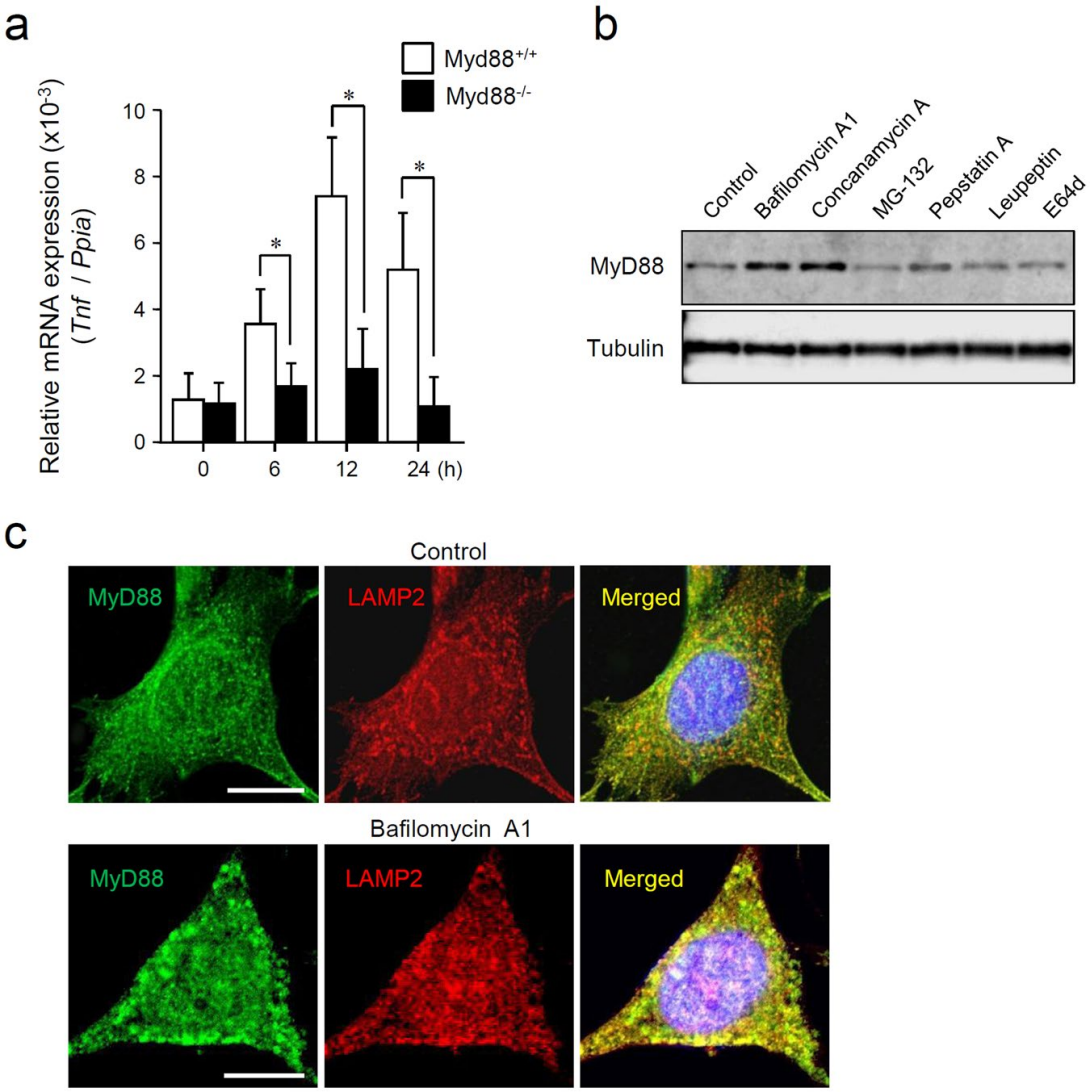
Lysosomal inhibition induces activation of MyD88-dependent inflammatory signals and accumulation of MyD88. (**a**) Deficiency of MyD88 impairs BafA1 treatment-induced *Tnf* expression. BMDMs collected from Myd88^+/+^ mice and Myd88^−/−^ mice were incubated with 100 nM BafA1 for the indicated periods, followed by RNA extraction. Relative expression levels of *Tnf* were determined by qRT-PCR. Each value is expressed as mean ± SD (n = 3); *p < 0.05 (Myd88^+/+^ versus Myd88^−/−^), one-way ANOVA and Dunnett’s test for post-hoc comparisons (μc ≠ μi). (**b**) Treatment with the inhibitors of lysosomal functions or lysosomal proteases increases basal MyD88 expression. Mouse BMDMs were incubated with 0.5% DMSO (control), 100 nM BafA1, 100 nM concanamycin A, 20 μM MG-132, 2 μM pepstatin A, 50 μM leupeptin, or 50 μM E-64-d for 12 h. Expression levels of MyD88 and α-tubulin were assessed by immunoblotting. All the blots were obtained under the same experimental conditions, and the cropped images of the blots are shown. The uncropped images are in Supplementary Fig. 18. (**c**) BafA1 treatment causes accumulation of MyD88. Mouse BMDMs were incubated with 0.5% DMSO (control) or 100 nM BafA1 for 6 h. The cells were fixed, and immunofluorescent staining for MyD88 (green) and LAMP2 (red) was carried out. Cell nuclei were stained with Hoechst 33342. Images were acquired by means of a confocal microscope. Scale bar: 10 μm.

BMDMs were isolated from wild-type (Myd88^+/+^) mice and *Myd88*-deficient (Myd88^−/−^) mice and treated with BafA1. Of note, *Tnf* expression in Myd88^−/−^ BMDMs was impaired as compared with that in Myd88^+/+^ cells (Fig. 2a). Such downregulation was also observed in *Il6* expression in Myd88^−/−^ MEFs (Supplementary Fig. 4). Thus, MyD88 is involved in generation of inflammatory signals during lysosomal impairment.

By analyzing the expression levels of MyD88, we found that BafA1 treatment increases the basal expression level of MyD88 (Fig. 2b and Supplementary Fig. 5). Upregulation of MyD88 was also observed after the treatment with concanamycin A or pepstatin A (Fig. 2b and Supplementary Fig. 5). Although MyD88 has been reported to be degraded in proteasomes after TLR signal transduction^19^, treatment of the cells with the proteasome inhibitor MG-132 did not influence the level of MyD88 (Fig. 2b and Supplementary Fig. 5). Next, MyD88 was visualized by immunofluorescence microscopy. Under normal conditions, MyD88 was distributed throughout the cytoplasm as small speckle-like structures (Fig. 2c, upper panels) consistent with other findings^20, 21^. These speckles hardly or only partly co-localized with the lysosome marker LAMP-2 (Fig. 2c, upper panels). On the other hand, during BafA1 treatment, MyD88 was obviously accumulated, and the bulk of MyD88 colocalized with LAMP-2 (Fig. 2c, lower panels). These observations suggest that the basal expression level of MyD88 is constitutively controlled by lysosomal proteolysis.

**Figure 3:**
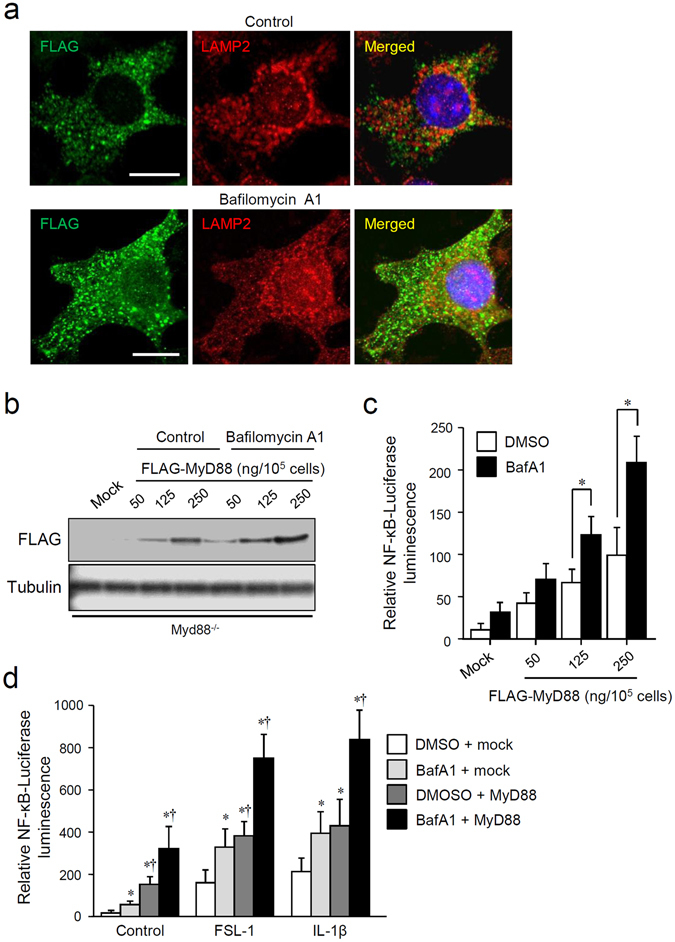
Lysosomal inhibition enhances activation of MyD88-dependent inflammatory signals via accumulation of MyD88. (**a**) BafA1 treatment increases expression of overexpressed MyD88. RAW264.7 cells were transfected with a plasmid encoding FLAG epitope-tagged MyD88, and then incubated with 0.5% DMSO (control) or 100 nM BafA1 for 12 h. The cells were fixed, and immunofluorescent staining for FLAG (green) and LAMP2 (red) was carried out. Cell nuclei were stained with Hoechst 33342. Images were captured by means of a confocal microscope. Scale bar: 10 μm. (**b**) BafA1 treatment increases the expression levels of overexpressed MyD88. RAW264.7 cells were transfected with the indicated amounts of a plasmid encoding FLAG epitope-tagged MyD88 or 250 ng/10^5^ cells of an empty pcDNA3 plasmid (mock), and then incubated with or without 100 nM BafA1 for 12 h. Expression levels of FLAG-MyD88 and α-tubulin were assessed by immunoblotting. All the blots were obtained under the same experimental conditions, and the cropped images of the blots are shown. The uncropped images are in Supplementary Fig. 19. (**c**) BafA1 treatment enhances overexpressed MyD88-induced NF-κB-dependent transcriptional activity. An NF-κB-driven luciferase reporter assay was performed on RAW264.7 cells transfected with a plasmid encoding FLAG epitope-tagged MyD88 or with 250 ng/10^5^ cells of an empty pcDNA3 plasmid (mock). The cells were incubated with 100 nM BafA1 for 12 h. Relative NF-κB activity was measured, and the data are expressed as mean ± SD (n = 3); *p < 0.05 (DMSO versus BafA1), one-way ANOVA and Dunnett’s test for post-hoc comparisons (μc < μi). (**d**) TLR2- or IL-1R-activated NF-κB-dependent transcriptional activity is augmented by accumulated MyD88. An NF-κB-driven luciferase reporter assay was performed on RAW264.7 cells transfected with 250 ng/10^5^ cells of a plasmid encoding FLAG epitope-tagged MyD88 (MyD88) or with an empty plasmid (mock). The cells were incubated with 0.5% DMSO or 100 nM BafA1 for 12 h, followed by incubation with or without 100 nM FSL-1 or 10 ng/ml IL-1β. Relative NF-κB activity was measured, and the data are expressed as mean ± SD (n = 3); *p < 0.05 (versus DMSO), Student’s *t* test, ^†^ *p* < 0.05 (versus DMSO), one-way ANOVA and Dunnett’s test for post-hoc comparisons (μc < μi).

We next investigated overexpression of MyD88, which was induced by plasmid transfection of FLAG epitope-tagged MyD88 into Myd88^−/−^ MEFs, and we studied the influence of lysosomal inhibition. In agreement with other reports^20, 22^, overexpressed MyD88 was present as condensed speckles or enlarged aggregates in the cytoplasm (Fig. 3a, upper panels). In BafA1-treated cells, overexpressed MyD88 accumulated further, and the amount of aggregates in the cytoplasm increased further (Fig. 3a, lower panels). Immunoblot analysis showed that BafA1 treatment increased the protein level of overexpressed MyD88, which was originally produced from the equivalent amount of a plasmid in untreated cells (Fig. 3b). The capacity of NF-κB activation by overexpressed MyD88 was then examined by lysosome inhibition. Overexpressed MyD88 activated transcriptional activity of NF-κB, whereas BafA1 treatment significantly augmented the activity of overexpressed MyD88 (Fig. 3c). Additionally, the activity of NF-κB in the cells stimulated with the TLR2 ligand FSL-1 or IL-1β was augmented by BafA1 treatment, and the activity was further enhanced by overexpression of MyD88 (Fig. 3d). These results suggest that intracellular accumulation of MyD88 during lysosomal impairment activates or enhances induction of inflammatory signals.

### MyD88 is targeted by basal autophagy that is different from macroautophagy

**Figure 4:**
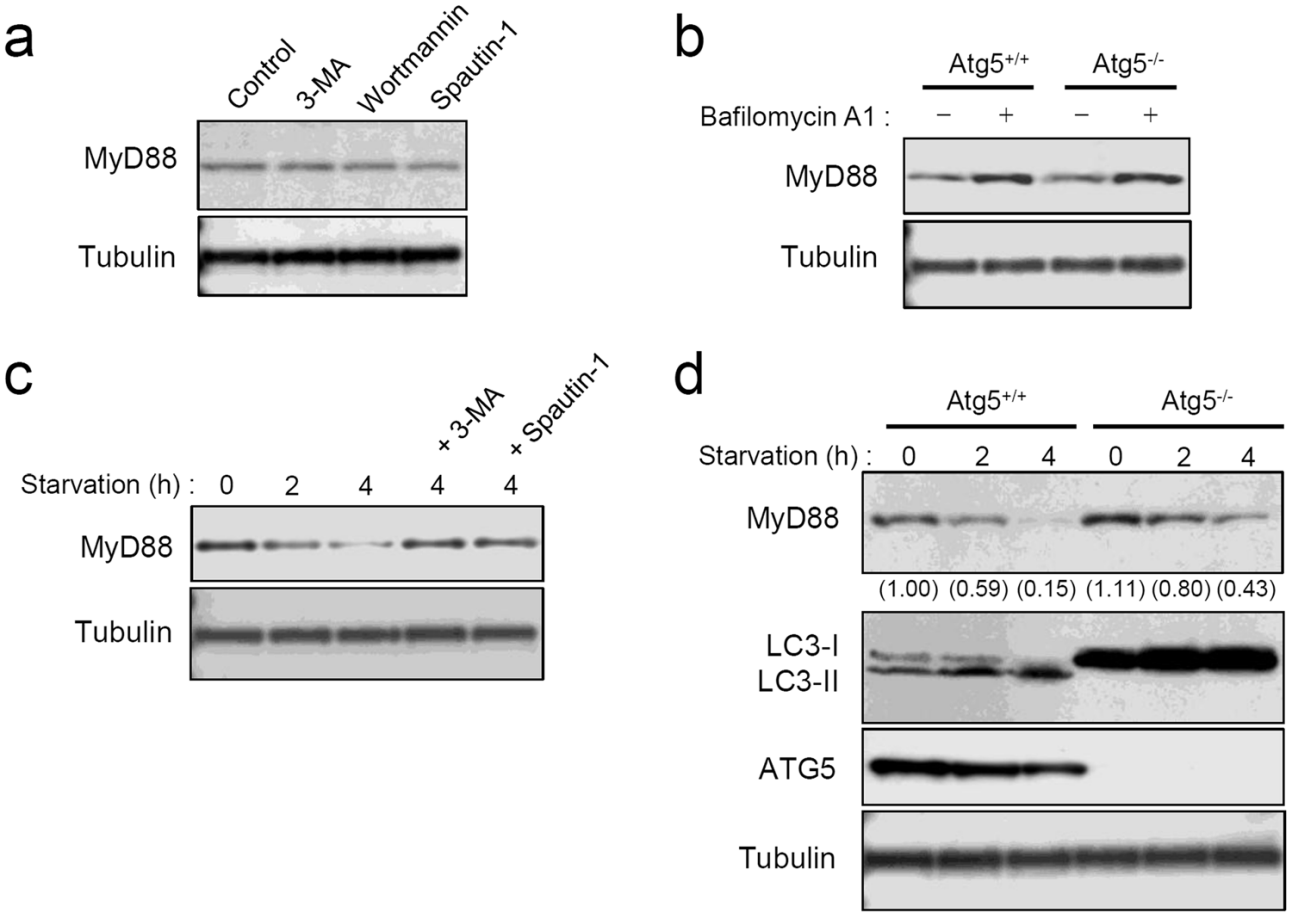
Basal autophagic degradation of MyD88 is mediated by a mechanism different from macroautophagy. (**a**) Treatment with the inhibitors of macroautophagy does not affect basal MyD88 expression. MEFs were incubated with 0.5% DMSO (control), 10 mM 3-MA, 100 nM wortmannin, or 10 μM spautin-1 for 12 h. Expression levels of MyD88 and α-tubulin were assessed by immunoblotting. All the blots were obtained under the same experimental conditions, and the cropped images of the blots are shown. The uncropped images are in Supplementary Fig. 20. (**b**) Deficiency in ATG5 does not affect BafA1 treatment-increased basal MyD88 expression. Atg5^+/+^ MEFs and Atg5^−/−^ MEFs were incubated with 0.5% DMSO (BafA1−) or 100 nM BafA1 (BafA1+) for 12 h. Expression levels of MyD88 and α-tubulin were assessed by immunoblotting. All the blots were obtained under the same experimental conditions, and the cropped images of the blots are shown. (**c**) Starvation-induced degradation of basal MyD88 is suppressed by the inhibitors of macroautophagy. MEFs were incubated with or without 10 mM 3-MA or 10 μM spautin-1 for 6 h, and then cultured under the starvation conditions for the indicated periods. Expression levels of MyD88 and α-tubulin were assessed by immunoblotting. All the blots were obtained under the same experimental conditions, and the cropped images of the blots are shown. (**d**) Starvation-induced degradation of basal MyD88 is affected by ATG5 deficiency. Atg5^+/+^ MEFs and Atg5^−/−^ MEFs were cultured under the starvation conditions for the indicated periods. Expression levels of MyD88, LC3, ATG5, and α-tubulin were assessed by immunoblotting. Values within parentheses represent the ratio determined by densitometric measurement of the bands. All the blots were obtained under the same experimental conditions, and the cropped images of the blots are shown. The uncropped images are in Supplementary Fig. 21.

The results described above revealed that MyD88 undergoes lysosomal proteolysis even in the presence of sufficient nutrients in the culture medium and even in the absence of any extracellular stimuli, suggesting that MyD88 is targeted by basal autophagy. To determine which type of autophagy is involved in this process, we tested general inhibitors of macroautophagy, 3-methyladenine (3-MA), wortmannin, and spautin-1, but none of them affected basal MyD88 expression (Fig. 4a and Supplementary Fig. 6). Additionally, in MEFs deficient in Atg5, an important component of macroautophagy^17^, the levels of basal MyD88 before or after BafA1 treatment were almost identical to the levels in Atg5^+/+^ MEFs (Fig. 4b and Supplementary Fig. 7). Furthermore, degradation of MyD88 was not affected by treatment with 6-aminonicotinamide, an activator of CMA^23^ (data not shown). Thus, the degradation of MyD88 by basal autophagy was assumed to be mediated by a process different from macroautophagy and CMA.

We next analyzed inducible autophagy in order to detect a possible difference from basal autophagy. Inducible autophagy was elicited by elimination of serum and amino acids from the culture medium. The expression of MyD88 was obviously decreased by nutrient elimination, but this decrease was attenuated by treatment with the macroautophagy inhibitors (Fig. 4c and Supplementary Fig. 8). Additionally, in both Atg5^+/+^ cells and Atg5^−/−^ cells, the expression of MyD88 gradually decreased after nutrient elimination, but the magnitude of the decrease in Atg5^−/−^ cells was moderate as compared with Atg5^+/+^ MEFs (Fig. 4d and Supplementary Fig. 9). Thus, inducible autophagy degrades MyD88 through both Atg5-dependent and -independent macroautophagy, which are likely to be the consequence of bulk degradation of the cytoplasm, and are obviously different from basal autophagy.

### Basal autophagy targets monomeric MyD88

**Figure 5:**
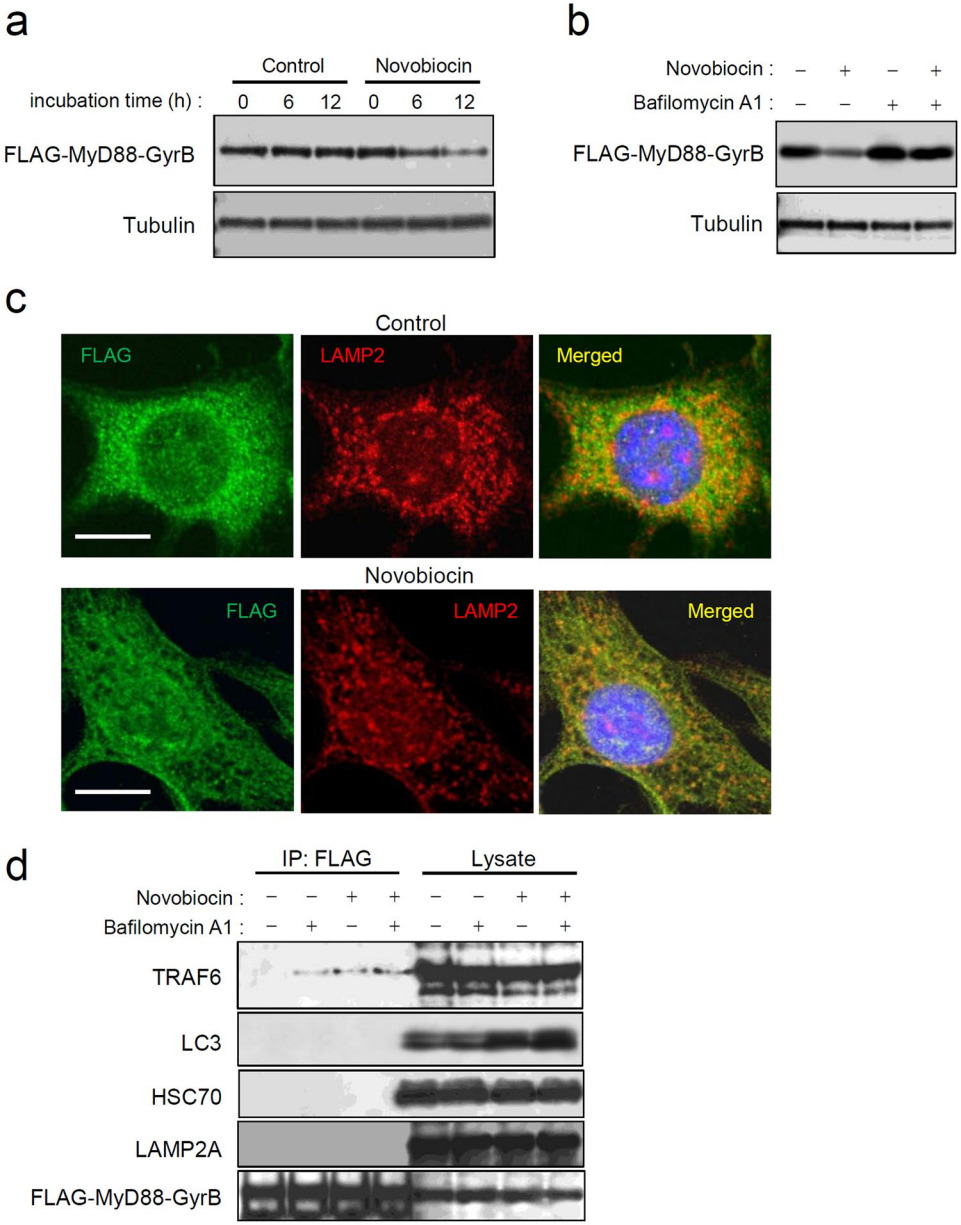
Monomeric MyD88 is targeted by basal autophagy. (**a**) Monomerization of MyD88-GyrB promotes its degradation. Myd88^−/−^ MEFs stably expressing FLAG-tagged MyD88-GyrB were incubated with 10 μM novobiocin for the indicated periods, followed by cell lysis. Expression levels of FLAG-MyD88-GyrB and α-tubulin were assessed by immunoblotting. All the blots were obtained under the same experimental conditions, and the cropped images of the blots are shown. The uncropped images are in Supplementary Fig. 22. (**b**) Degradation of monomerized MyD88-GyrB is inhibited by lysosomal inhibition. Myd88^−/−^ MEFs stably expressing FLAG-tagged MyD88-GyrB were incubated with or without 10 μM novobiocin for 12 h in the presence or absence of 100 nM BafA1. Expression levels of FLAG-MyD88-GyrB and α-tubulin were assessed by immunoblotting. All the blots were obtained under the same experimental conditions, and the cropped images of the blots are shown. (**c**) Speckles of MyD88-GyrB are downregulated by monomerization. Myd88^−/−^ MEFs stably expressing FLAG-tagged MyD88-GyrB were incubated with 0.5% DMSO or 10 μM novobiocin for 12 h. Immunofluorescent staining of the cells for FLAG (green) and LAMP2 (red) was carried out, and cell nuclei were stained with Hoechst 33342. Images were obtained by confocal microscopy. Scale bar: 10 μm. (**d**) Monomerized MyD88-GyrB interacts with TRAF6. Myd88^−/−^ MEFs stably expressing FLAG-tagged MyD88-GyrB were incubated with or without 10 μM novobiocin for 12 h in the presence or absence of 100 nM BafA1, followed by cell lysis. Then immunoprecipitation (IP) using anti-FLAG-agarose was carried out with clarified lysates, followed by immunoblotting for TRAF6, p62/Sqstm1, LC3, ATG5, HSC70, LAMP2A, and FLAG-MyD88-GyrB. All the blots were obtained under the same experimental conditions, and the cropped images of the blots are shown.

Inactive MyD88 is thought to exist as monomers and/or dimers in the cytoplasm^20, 24^. We tested whether the molecular state of MyD88 affects its degradability. To this end, we introduced a chimeric MyD88 protein that has a C-terminal fusion with the bacterial DNA gyrase B subunit (MyD88-GyrB). The GyrB portion has two important roles: to prevent autoactivation of MyD88 and to make it possible for us to force monomerization via binding with novobiocin as a 1:1 complex^25^. In Myd88^−/−^ MEFs stably expressing N-terminal FLAG epitope-tagged MyD88-GyrB, this construct was present as small speckles in the cytoplasm, similarly to endogenous MyD88 speckles (Supplementary Fig. 10). Despite the overexpressed state, it did not trigger any inflammatory signals, such as NF-κB activation (data not shown). This observation indicates that the speckles of MyD88 were inactive and able to resist basal autophagic degradation even if the amount of the speckles was superabundant. Moreover, treatment with novobiocin, which enables monomerization of MyD88-GyrB^25^, induced degradation of MyD88-GyrB (Fig. 5a and Supplementary Fig. 11). Monomerization-induced degradation of MyD88-GyrB was attenuated by BafA1 treatment (Fig. 5b). Microscopic visualization revealed that novobiocin treatment reduced the number of the speckles and the expression level of MyD88-GyrB (Fig. 5c). These results suggest that monomeric MyD88 is preferentially targeted by basal autophagy.

Consequently, we hypothesized the existence of a factor that enables stabilization of MyD88 speckles. To test this theory, Myd88^−/−^ MEFs stably expressing FLAG-tagged MyD88-GyrB were treated with novobiocin in the presence or absence of BafA1, followed by co-immunoprecipitation with anti-FLAG antibody-conjugated agarose. BafA1 treatment induced interaction with TRAF6 (Fig. 5d), indicating that lysosomal inhibition actually activates MyD88- and TRAF6-mediated inflammatory signals. Unexpectedly, novobiocin treatment also induced MyD88-GyrB interaction with TRAF6 (Fig. 5d), indicating that monomeric MyD88 also interacts with TRAF6. The magnitude of TRAF6 interaction with monomeric MyD88-GyrB was hardly influenced by BafA1, which noticeably increased the cellular level of LC3 in the lysates (Fig. 5d), suggesting that TRAF6-interacting MyD88 is protected from autophagic degradation. Nevertheless, an interaction with macroautophagy marker LC3 was not observed even during BafA1 treatment (Fig. 5d). Additionally, an interaction with the markers of CMA Hsc70/Hspa8 and LAMP-2A was not observed (Fig. 5d). Thus, TRAF6 may participate in protection of monomeric MyD88 from targeting by basal autophagy. Moreover, essential molecules required for macroautophagy and CMA do not interact with monomeric MyD88 during basal autophagic processes.

### TRAF6 serves as a stabilizing factor for MyD88

**Figure 6:**
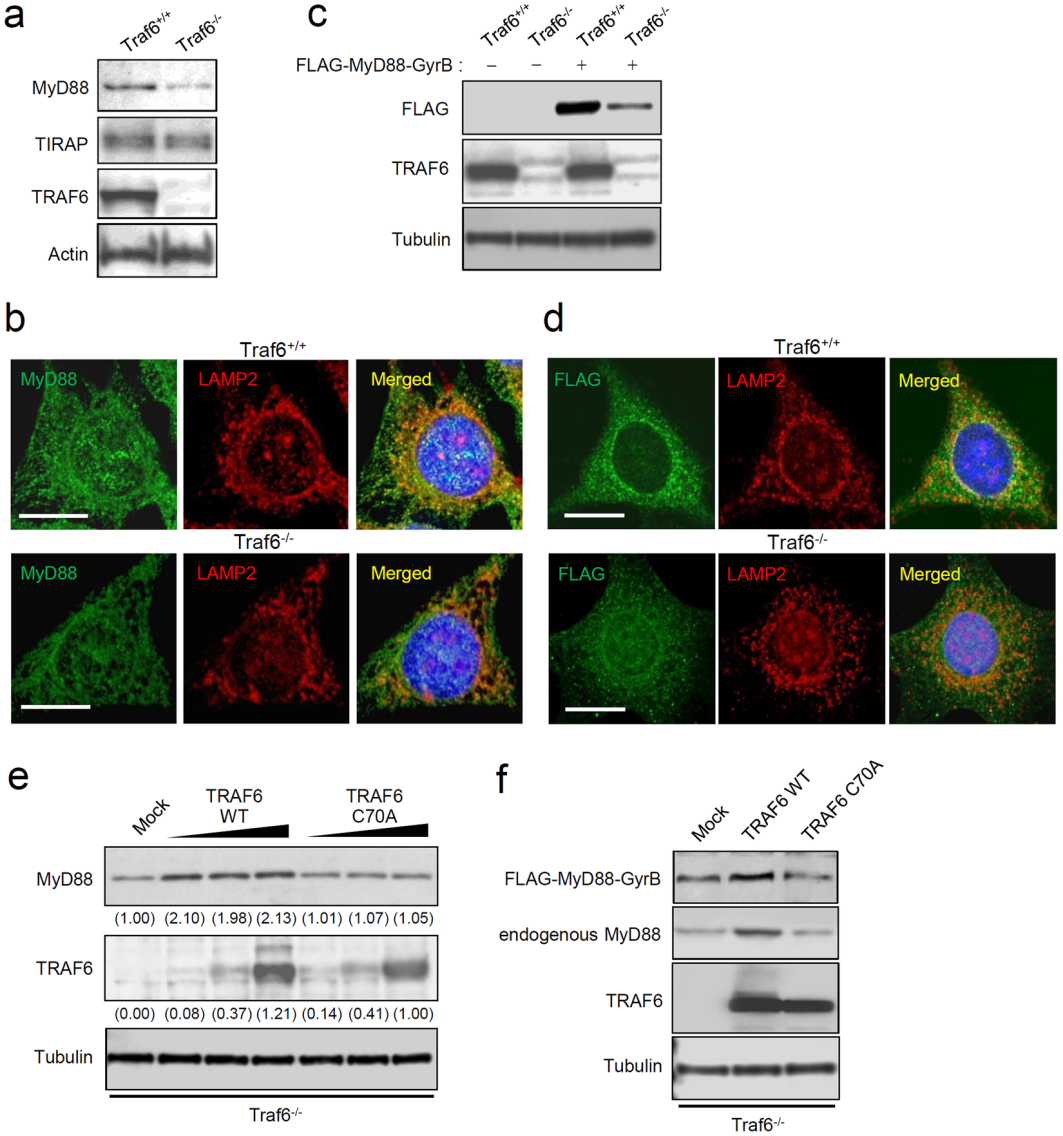
TRAF6 facilitates resistance of MyD88 to degradation. (**a**) Basal MyD88 expression is lowered by TRAF6 deficiency. Expression of MyD88, TIRAP, TRAF6, and actin in Traf6^+/+^ MEFs and Traf6^−/−^ MEFs was assessed by immunoblotting. All the blots were obtained under the same experimental conditions, and the cropped images of the blots are shown. The uncropped images are in Supplementary Fig. 23. (**b**) MyD88 speckles are downregulated by TRAF6 deficiency. Immunofluorescent staining of Traf6^+/+^ MEFs and Traf6^−/−^ MEFs for MyD88 (green) and LAMP2 (red) was carried out. Cell nuclei were stained with Hoechst 33342. Images were captured by means of a confocal microscope. Scale bar: 10 μm. (**c**) Stably expressed MyD88-GyrB is downregulated by TRAF6 deficiency. Traf6^+/+^ MEFs and Traf6^−/−^ MEFs were stably transfected with a plasmid encoding FLAG-MyD88-GyrB or with an empty plasmid. Expression of MyD88, TRAF6, and α-tubulin was assessed by immunoblotting. All the blots were obtained under the same experimental conditions, and the cropped images of the blots are shown. The uncropped images are in Supplementary Fig. 24. (**d**) Speckles of MyD88-GyrB are downregulated by TRAF6 deficiency. Traf6^+/+^ MEFs and Traf6^−/−^ MEFs were stably transfected with the FLAG-MyD88-GyrB construct. Immunofluorescent staining for FLAG (green) and LAMP2 (red) was carried out, and cell nuclei were stained with Hoechst 33342. Images were acquired by means of a confocal microscope. Scale bar: 10 μm. (**e**) Basal MyD88 in TRAF6-deficient cells is restored by TRAF6. Traf6^−/−^ MEFs were transiently transfected with increasing amounts of a plasmid encoding FLAG-tagged wild-type TRAF6 (TRAF6 WT) or enzymatically inactive TRAF6 (TRAF6 C70A) or with an empty plasmid (mock). Expression of MyD88, FLAG-TRAF6, and α-tubulin was assessed by immunoblotting. Values within parentheses represent the ratio determined by densitometric measurement of the bands. All the blots were obtained under the same experimental conditions, and the cropped images of the blots are shown. The uncropped images are in Supplementary Fig. 25. (**e**) MyD88-GyrB expressed in TRAF6-deficient cells is restored by TRAF6. Traf6^−/−^ MEFs stably expressing FLAG-MyD88-GyrB were transiently transfected with a plasmid encoding TRAF6 WT or TRAF6 C70A, or an empty plasmid (mock). Expression of FLAG-MyD88-GyrB, endogenous MyD88, TRAF6, and α-tubulin was assessed by immunoblotting. All the blots were obtained under the same experimental conditions, and the cropped images of the blots are shown.

We next focused on the role of TRAF6 in the basal autophagic degradation of monomeric MyD88. An experiment with a *Traf6* knockdown by small interfering RNA (siRNA) revealed that the basal expression level of MyD88 protein is lowered along with downregulation of TRAF6 (Supplementary Fig. 12). The *Traf6* knockdown did not affect the transcription of *Myd88* (Supplementary Fig. 13). *Traf6*-deficient cells were further analyzed, and the basal expression level of MyD88 was found to be noticeably decreased in Traf6^−/−^ MEFs as compared with Traf6^+/+^ MEFs (Fig. 6a and Supplementary Fig. 14). On the other hand, the basal expression level of another TLR adaptor protein, TIRAP (also known as Mal), was almost identical between these cell lines (Fig. 6a). Incidentally, *Traf6* deficiency did not affect the transcription of *Myd88* (Supplementary Fig. 15). Microscopic analysis revealed that small speckles of MyD88 are appreciably downregulated in Traf6^−/−^ MEFs (Fig. 6b). Stable expression of FLAG-tagged MyD88-GyrB in these MEFs revealed that its basal expression level in Traf6^−/−^ MEFs was lowered compared with that in Traf6^+/+^ MEFs (Fig. 6c). Microscopic analysis of Traf6^−/−^ MEFs stably expressing MyD88-GyrB showed that the amount of the speckles was lowered (Fig. 6d). These results suggest that TRAF6 serves as a stabilizing factor of MyD88 by mediating formation of speckles although TRAF6 deficiency does not eliminate the speckles or basal expression of MyD88.

We then determined whether the enzymatic activity of TRAF6 is implicated in the stabilization of MyD88. We prepared mutated TRAF6 (C70A) that lacks the E3 ubiquitin ligase activity^26^ and a plasmid encoding its FLAG epitope-tagged protein was transfected into Traf6^−/−^ MEFs. We found that wild-type TRAF6 restored the basal expression level of endogenous MyD88 (Fig. 6e). The maximal level of MyD88 was limited despite the presence of increasing amounts of the plasmid encoding TRAF6 (Fig. 6e), suggesting that overexpressed TRAF6 does not stimulate the transcription of *Myd88*. Of note, TRAF6 mutant C70A could not restore basal MyD88 expression (Fig. 6e). Furthermore, in Traf6^−/−^ MEFs stably expressing MyD88-GyrB, transfection of the plasmid encoding wild-type TRAF6, but not TRAF6 C70A, led to restoration of the expression level of MyD88-GyrB (Fig. 6f). Thus, the E3 ubiquitin ligase activity of TRAF6 is essential for the stabilization of basal MyD88. Conversely, in the absence of TRAF6, MyD88 may lapse into the unstable state susceptible to degradation by basal autophagy.

## Discussion

Our results indicated that lysosomal impairment weakly activates inflammatory signals, including activation of transcription factors NF-κB and AP-1 and transcription of proinflammatory cytokines. This effect was accompanied by intracellular accumulation of MyD88, which autoactivated downstream signaling and enhanced signaling of TLRs and IL-1R. These observations essentially indicated that accumulation and subsequent autoactivation of MyD88 are intrinsically prevented. This study revealed that this effect is mediated by basal autophagy, which seems different from typical macroautophagy and CMA. Basal autophagy was found to selectively and preferentially degrade monomeric MyD88, while MyD88 speckles, which are probably a dimerized or oligomerized form of MyD88, can resist basal autophagic targeting. Moreover, the degradability of MyD88 was found to be regulated by the enzymatic activity of TRAF6. Namely, monomeric MyD88 that is not interacting with TRAF6 seems to be preferentially degraded. Overall, basal autophagy prevents inflammatory signals through regulation of basal MyD88 expression, but, under conditions of a failure of lysosomal proteolysis, inflammatory signals are elicited by accumulated MyD88. Such signals may influence various parameters of inflammatory conditions and may be especially important as initial inflammatory signaling for production of pro-IL-1β prior to activation of secondary inflammatory signaling for inflammasome activation.

Other studies on the subcellular localization have shown that overexpressed or activated MyD88 forms large aggregates or inclusion bodies^20, 22, 27^. We previously demonstrated that formation of such aggregates is associated with the interaction of MyD88 with Sqstm1/p62 and HDAC6^20^, both of which are ubiquitin-binding molecules that facilitate macroautophagic targeting of protein aggregates^28^. Another study group reported that MyD88 aggregates can be degraded by macroautophagy^29^. On the other hand, it was previously unknown whether autophagy is involved in the degradation of inactive MyD88. Our present results indicate that inactive monomeric MyD88 is degraded by basal autophagy, whose mechanism is different from that of macroautophagy. This is because general macroautophagy inhibitors and Atg5 deficiency did not affect this degradation (Fig. 4a and b). This autophagy type is also different from CMA because the CMA activator 6-aminonicotinamide did not affect MyD88 degradation, and MyD88 did not interact with the essential molecules Hsc70/Hspa8 and LAMP-2A (Fig. 5d). Additionally, MyD88 does not carry any typical conserved KFERQ-related motifs that are required for CMA^17, 30^. Thus, there is high likelihood that this autophagy corresponds to microautophagy^17^.

MyD88 serves as a pivotal signaling adaptor for TLRs, IL-1R, and IL-18 receptor (IL-18R)^3, 31^. MyD88 consists of two functional domains, an N-terminal death domain and a C-terminal Toll/IL-1 receptor homology domain, with an intermediate linker region^31^. After receptor ligation, MyD88 transiently forms a homohexamer that recruits four molecules of IL-1 receptor-associated kinase (IRAK) 1 or IRAK2, and subsequently recruits four molecules of IRAK4, ultimately forming the complex termed ‘myddosome’ for activation of TRAF6-mediated signaling^31^. Nevertheless, it was previously unclear how MyD88 is kept inactive or how the basal expression level of MyD88 can be controlled. It was also unknown how overexpressed MyD88 autoactivates downstream signaling even in the absence of receptor ligation although this phenomenon had been known for a long time^18^. In the present study, we uncovered two important mechanisms: i) the TRAF6-mediated stability control of basal MyD88 and ii) degradation of monomeric MyD88 by basal autophagy. TRAF6 is involved in the subcellular localization of basal MyD88, i.e. formation of small speckles that are probably a stable and inactive form of MyD88 (Fig. 6). Most of MyD88 that has not been assisted by TRAF6 is likely unstable and is destined for degradation by basal autophagy. This way of lowering the basal MyD88 expression may serve as a preparative mechanism against induction of inflammatory signals of TLRs, IL-1R, and IL-18R, lowering the threshold of cellular sensitivity to these inflammatory signals. Conversely, stable small speckles may be activated only after receptor ligation. The detailed mechanism behind the activation or inactivation of MyD88 small speckles should be fully explored in future studies.

TRAF6 has two different functions: (i) an E3 ubiquitin ligase that works together with the complex of Ubc13 and Uev1A or with Mms2 and (ii) a scaffold protein that mediates formation of a signaling complex. Due to these functions, TRAF6 serves as a ubiquitin-dependent signal transducer, ultimately activating transcription factors NF-κB and AP-1 downstream of TLRs, IL-1R, and various cytokine receptors^3^. TRAF6 also has roles in autophagy, which may be unrelated to these signal transduction. For example, performing an mTOR-associated function, TRAF6 interacts with AMBRA1 to ubiquitinate ULK1, leading to suppression of autophagy induction^32^. Conversely, TRAF6 induces ROS-mediated autophagy through interaction with Atg9^33^. In the present study, we provided new evidence that TRAF6 serves as a factor that regulates the degradability of monomeric MyD88 that is targeted by basal autophagy. The results from *Traf6*-deficient cells suggest that TRAF6 is implicated in stabilization of MyD88 (Fig. 6a–d). TRAF6 seems to interact with only a part of the monomeric-MyD88 pool to switch it to small speckles that acquire protection from basal autophagic degradation. This activity is completely dependent on the E3 ubiquitin ligase activity of TRAF6 (Fig. 6e and f). In *Traf6*-deficient cells, the number of MyD88 speckles was considerably reduced, but not zero (Fig. 6b), suggesting that another factor compensates for the loss of TRAF6. TRAF3 may be a candidate because it serves as an E3 ligase acting downstream of MyD88^25^.

Lysosomes fuse with other vesicular organelles, such as late endosomes, phagosomes, and autophagosomes, and virtually disassimilate most of biomolecules, including peptides, nucleic acids, and carbohydrates, that are engulfed by the cell or generated inside the cell; thereby lysosomes are involved in various cellular processes^34^. Because a failure in the functioning of lysosomes can be induced in various settings, including genetic factors^35, 36^, oxidative stress^37^, and mitochondrial dysfunction^38^, increasing evidence indicates that lysosomal impairment is associated with activation of inflammatory signals^9, 39^. For instance, impairment of the autophagy–lysosome pathway causes dysfunction of mitochondrial clearance, which results in generation of ROS and a release of mitochondrial DNA^9^. Furthermore, destabilized lysosomes release cathepsin B^40^. These phenomena lead to activation of NLRP3-mediated inflammatory signaling and formation of inflammasomes. Although inflammasomes activate caspase-1 to cleave pro-IL-1 and pro-IL-18, production of these precursor cytokines requires initial signaling such as NF-κB signaling. Our results suggest that impairment of lysosomes, especially of lysosomal aspartic proteases, causes MyD88 accumulation that triggers such initial inflammatory signals. MyD88-mediated signaling has been actually shown to be important for the production of pro-IL-1β and pro-IL-18^3, 41^. It is also known that MyD88-mediated signaling incidentally exacerbates inflammatory conditions, and its excessive or prolonged induction leads to chronic inflammation associated with autoimmunity and autoinflammation^42, 43^. Thus, in addition to the significance of NLRP3 inflammasomes, autoactivation of MyD88 may participate in induction of inflammatory responses when autophagy-lysosome pathways are dysfunctional.

We demonstrated that NF-κB activation induced by a TLR2 ligand or IL-1β is enhanced during MyD88 accumulation and lysosomal impairment (Fig. 2d). Given that MyD88 is ubiquitously expressed, accumulated-MyD88-induced autoactivation of inflammatory signals may be found in various types of cells under conditions of lysosomal impairment. MyD88-elicited inflammatory signals can be concomitantly enhanced by other circumstantial conditions. For instance, IL-1β and IL-18 are generated by the cleavage of pro-IL-1β and pro-IL-18 by activated inflammasomes; these molecules further activate MyD88-dependent signaling through IL-1R and IL-18R^44^. Alternatively, under conditions of excessive generation of ROS, accumulation of tissue damage triggers production of endogenous activators of innate immunity known as DAMPs^45, 46^. Various kinds of DAMPs stimulate TLRs, especially TLR2 and TLR4, to activate MyD88-dependent signaling^3, 47^. Importantly, these responses, including activation of inflammasomes, generation of ROS, and transduction of MyD88-dependent signals, have been reported to be downregulated by autophagy^8, 11, 48, 49^. Nevertheless, most of the reported effects of autophagy work along with or after the induction of inflammatory signals as inducible macroautophagy, and they are exerted directly on signaling inducers. On the other hand, our present results showing prevention of inflammatory signals by basal autophagic degradation of MyD88 represent an initial and indirect effect. Thus, these observations imply that the onset of inflammation associated with a failure in the function of lysosomes or autophagy involves a more comprehensive set of failures in the regulatory effects of both basal and inducible autophagy.

Dysfunction of lysosomes or autophagy is associated not only with autoimmunity and autoinflammation but also with the inflammatory status of aging (termed inflamm-aging)^9, 10, 50, 51^. Aging processes gradually cause a decline of autophagic cleansing capability, thus leading to impaired turnover of mitochondria and of detrimental proteins. These events lead to activation of NLRP3 inflammasomes and production of IL-1β and IL-18, further accelerating aging processes by driving the development of neurodegenerative diseases, cardiovascular diseases, and systemic low-grade inflammation^51, 52^. Although other reports have mainly been focused on NLRP3 inflammasomes, our results show the additional implication of MyD88-mediated signaling. It is possible that an aging-related decline of lysosomal functions induces chronic accumulation of MyD88 and affects chronic inflammatory status in the elderly. Additionally, given that MyD88 is an aggregation-prone protein^20, 53^, it is possible that chronic accumulation of MyD88 promotes neurodegenerative disorders. Alternatively, accumulated MyD88 may augment inflammatory responses against PAMPs, DAMPs, IL-1β, and IL-18. Thus, future clinical studies should verify the significance of MyD88 accumulation during the impairment of lysosomal or autophagic functions.

## Materials and Methods

### Reagents

BafA1 was obtained from Wako Chemicals or AdipoGen Life Sciences. Concanamycin A was obtained from Santa Cruz Biotechnology. Pepstatin A, E-64-d and leupeptin were obtained from Peptide Institute (Osaka, Japan). Wortmannin and MG-132 were obtained from Calbiochem. Ammonium chloride, novobiocin sodium salt, 3-MA, spautin-1 and 6-aminonicotinamide were obtained from Sigma. Recombinant mouse IL-1β was obtained from Cell Signaling Technology. The TLR2 ligand FSL-1 was obtained from InvivoGen. Other reagents were obtained from Wako Chemicals.

### Cells

Murine bone marrow-derived macrophages (BMDMs) were prepared from bone marrow hematopoietic cells isolated from femurs and tibias of male B6J Jms Slc mice and B6 background *Myd88* deficient mice (Myd88^−/−^ mice)^54^. All animal experiments were approved by the Animal Experimental Committees of Asahi University School of Dentistry (Permit Numbers: 14-006, 15-001, and 16-008) and were carried out in accordance with the approved guidelines. Dispersed hematopoietic cells in the basal medium (RPMI 1640 supplemented with 10% of heat-inactivated fetal bovine serum (FBS; Gibco), 50 μM 2-mercaptoethanol, 100 U/ml penicillin, 100 μg/ml streptomycin, and 50 μg/ml gentamycin) were filtered through a 70-μm cell strainer (BD Biosciences) and then treated with hemolytic BD Pharm Lyse buffer (BD Biosciences) followed by washing with the medium. Cells were resuspended in the differentiation medium (basal medium supplemented with 40 ng/ml M-CSF (Affymetrix)), and the cells at the concentration of 5 × 10^5^/ml in 10 ml of the differentiation medium were seeded in 100-mm diameter culture dishes for incubation for 2 days. On day 3, 5 ml of the fresh differentiation medium was added, with cultivation for additional 2 days. Nonadherent cells were aspirated, and adherent cells were gently detached using Sumilon M cell scrapers (Sumitomo Bakelite, Tokyo, Japan) in the presence of 5 ml of the differentiation medium. The collected cells were plated in 24-well culture plates (at 10^5^/ml) and incubated overnight to obtain adherent macrophages for the use in experiments. MEFs were prepared from 13.5-day embryos of B6 mice as described elsewhere^55^, and these cells were used within three passages. Atg5^−/−^ MEFs and control Atg5^+/+^ MEFs originally established by N. Mizushima^56^ were obtained from RIKEN BRC Cell Bank (Tsukuba, Japan). Traf6^−/−^ MEFs and control Traf6^+/+^ MEFs were described previously^57^. MEFs were maintained in Dulbecco’s Modified Eagle’s Medium (DMEM) supplemented with 10% FBS, 2 mM L-glutamine, sodium pyruvate, nonessential amino acids, 100 U/ml penicillin, and 100 μg/ml streptomycin. RAW264.7 cells were obtained from RIKEN BRC Cell Bank and maintained as described previously^20^. All the cells were cultivated at 37 °C in the atmosphere containing 5% of CO_2_.

### Plasmids, siRNA, and transfection

The expression plasmid for FLAG epitope-tagged mouse TRAF6 was described previously^48^. The constructs encoding the FLAG epitope-tagged enzyme-inactive mutant of TRAF6 (Cys70 substituted with Ala; lacking the E3 ubiquitin ligase activity^58^) was generated using a PrimeSTAR Mutagenesis Basal Kit (TaKaRa, Shiga, Japan). The expression plasmid for FLAG epitope-tagged mouse MyD88 C-terminally fused to the *E*. *coli* DNA gyrase B subunit was described elsewhere^20, 24, 25^. Transfection of plasmids was performed by means of the X-tream Gene HP DNA transfection reagent (Roche Applied Science). Traf6^+/+^ MEFs and Traf6^−/−^ MEFs stably transfected with the construct encoding FLAG-MyD88-GyrB were maintained in DMEM supplemented with 10% of FBS and 1 mg/ml G418 (Roche Applied Science). Stealth predesigned siRNA against mouse *Traf6* (MSS212084) and the control non-targeting siRNA (negative control med GC; 12935-300) were acquired from Thermo Fisher Scientific. The Lipofectamine RNAiMAX reagent (Thermo Fisher Scientific) and Opti-MEM I medium (Thermo Fisher Scientific) were used to transfect 100 nM of siRNA, according to the manufacturer’s instructions. After 24 h of incubation, culture media were changed to DMEM supplemented with 10% of FBS, and the cells were incubated until use in experiments.

### Quantitative reverse-transcription polymerase chain reaction (qRT-PCR)

Total RNA was extracted from cultured cells using the PureLink RNA Mini Kit (Thermo Fisher Scientific) and reverse-transcribed using ReverTra Ace qPCR RT Master Mix with the gDNA Remover Kit (TOYOBO). SYBR Green-based qRT-PCR was performed using SsoFast EvaGreen Supermix (Bio-Rad) and the Thermal Cycler Dice Real-Time System TP800 (TaKaRa). The designed primer sets for mouse *Tnf*, *Il1b*, *Il6*, *Myd88*, and *Ppia* were purchased from TaKaRa. The assessment of gene expression was carried out by the ΔΔC_t_ method. Results shown as relative expression were normalized to the levels of housekeeping gene *Ppia* and are representative of three independent experiments.

### A luciferase reporter gene assay for determination of the activities of NF-κB and AP-1

A dual luciferase reporter gene assay for the measurement of the firefly luciferase under the control of an NF-κB-driven or AP-1-driven promoter and the *Renilla* luciferase under the control of a constitutively active thymidine kinase promoter was performed as described previously^55, 59^. Each luciferase was activated using a Dual-Luciferase Reporter Assay System (Promega), and chemiluminescence was measured using 96F white microwell SI plates (Thermo Fisher Scientific) and an Infinite M200 PRO plate reader (TECAN). The results expressed as relative luminescence (firefly luciferase/*Renilla* luciferase) are representative of at least three independent experiments.

### Immunoblotting

Cell lysates were prepared using lysis buffer consisting of 20 mM HEPES (pH 7.4), 1% Triton X-100, 0.5% Nonidet P-40, 150 mM sodium chloride, 12.5 mM β-glycerophosphate, 1.5 mM magnesium chloride, 10 mM sodium fluoride, 2 mM DTT, 1 mM sodium orthovanadate, 2 mM EGTA, 1 mM phenylmethylsulfonyl fluoride, EDTA-free Complete protease inhibitor mixtures (Roche Applied Science), and PhosSTOP phosphatase inhibitor mixtures (Roche Applied Science) for 15 min at 4 °C. The collected lysates were centrifuged, and the supernatants were boiled with SDS sample buffer, and subjected to SDS-polyacrylamide gel electrophoresis (10–20% gradient gel) under reducing conditions. Separated proteins in gels were transferred to Immobilon-P transfer membranes (Millipore) using EzFastBlot buffer (ATTO; Tokyo, Japan) and a Trans-Blot Turbo transfer device (Bio-Rad; constant 25 V for 10 min). The membranes were blocked in EzBlockChemi buffer (ATTO) for 45 min. Immunoreactive bands were detected using the following primary antibodies and a horseradish peroxidase-conjugated secondary antibody: anti-MyD88 (D80F5) rabbit monoclonal antibody (mAb), anti-TIRAP (D6M9Z) rabbit mAb, anti-LC3A/B (D3U4C) rabbit mAb, anti-SQSTM1/p62 rabbit polyclonal Ab (pAb; 5114), anti-Atg5 (D5F5U) rabbit mAb, anti-HSPA8 (D12F2) rabbit mAb, and anti-α-tubulin (11H10) rabbit mAb obtained from Cell Signaling Technology; anti-TRAF6 rabbit polyclonal Ab (pAb) and anti-FLAG M2 mouse mAb obtained from SIGMA; anti-LAMP-2A rabbit pAb (51-2200) obtained from Thermo Fisher Scientific; anti-Actin (H-196) obtained from Santa Cruz Biotechnology. The Clarity Western ECL Substrate (Bio-Rad) was used to visualize the blots on an ECL minicamera (Amersham Biosciences) with the instant black and white film FP-3000B (Fuji Films). The immunoblot images were obtained using a CanoScan LiDE 40 scanner (Canon), and densitometric quantification of the immunoblot bands was performed in the ImageJ densitometry software (version 1.6, National Institutes of Health). In each experiment, blots for α-tubulin were shown as loading controls.

### Immunofluorescence and microscopic analysis

For immunofluorescence microscopy of BMDMs and MEFs, cells (5 × 10^4^/well) were seeded on Lab-Tek chamber 8-well Permanox slides (Nunc) and fixed at −20 °C with methanol for 15 min. Double immunofluorescent staining was then carried out using an anti-MyD88 (F-19) goat pAb (Santa Cruz Biotechnology) and Alexa 488-conjugated anti-goat IgG antibody (Thermo Fisher Scientific) and then with an anti-LAMP-2 rabbit pAb (Proteintech, 10397-1-AP) and Alexa 564-conjugated anti-rabbit IgG antibody (Thermo Fisher Scientific). For immunofluorescent staining of cells expressing FLAG epitope-tagged MyD88, an anti-FLAG M2 mouse mAb and Alexa 488-conjugated anti-mouse IgG antibody (Thermo Fisher Scientific) were used instead. Cell nuclei were also stained with Hoechst 33342 (Thermo Fisher Scientific), and the stained cells were embedded in the presence of the Prolong Gold Antifade reagent (Thermo Fisher Scientific). Images were captured using an LSM 710 confocal microscope system (Carl Zeiss).

### Immunofluorescence-based quantification of MyD88 expression

Cells (5 × 10^5^/well) were cultured in lumox multiwell 24 plates (Sarstedt). After the chemical treatment, the cells were fixed at −20 °C with methanol for 15 min, and blocked with 0.5% BSA in PBS for 30 min. Immunofluorescence was performed using an anti-MyD88 goat pAb and Alexa 488-conjugated anti-goat IgG antibody for staining of endogenous MyD88, or using an anti-FLAG M2 mouse mAb and Alexa 488-conjugated anti-mouse IgG antibody for staining FLAG epitope-tagged MyD88. Optimized fluorescence intensity of each well was measured using an Infinite M200 PRO plate reader in the presence of the Prolong Gold Antifade reagent, and the results were expressed as the average of nine spots per well. Results are representative of at least three independent experiments.

### Immunoprecipitation

The immunoprecipitation of FLAG-tagged MyD88-GyrB using the clarified lysates of Traf6^−/−^ MEFs was performed according to the protocol described elsewhere^20^.

### Statistical analysis

In the results of Figs 1a–c, 2a, 3c and d, and Supplementary Figs 1–7 and 17, data expressed as mean ± standard deviation (SD), were analyzed using one-way factorial analysis of variance (ANOVA) followed by Dunnett’s multiple (pairwise) comparison test. In the results of Supplementary Figs 8, 9 and 11, data were analyzed using two-way factorial ANOVA followed by Dunnett’s multiple tests for comparison between the groups of interest. In the results of Fig. 3d and Supplementary Figs 13 and 15, *p* values were calculated using Student’s *t* test. A two-tailed *p* value < 0.05 was considered significant.

## Electronic supplementary material

Supplementary Figures
