# Supplementary material for: Basal autophagy prevents autoactivation or enhancement of inflammatory signals by targeting monomeric MyD88
Source: Sci Rep. 2017 Apr 21;7:1009. doi: 10.1038/s41598-017-01246-w (PMC5430896; doi:10.1038/s41598-017-01246-w)
Supplement: Supplementary file 1 — Supplementary Figures [file 41598_2017_1246_MOESM1_ESM.pdf]

Supplementary Information

Basal autophagy prevents autoactivation or enhancement of inflammatory signals by targeting monomeric MyD88

Takeshi Into[1]*, Toshi Horie[1], Megumi Inomata[1], Jin Gohda[2], Jun-ichiro Inoue[3], Yukitaka Murakami[1] & Shumpei Niida[4]

[1]Department of Oral Microbiology, Division of Oral Infections and Health Sciences, Asahi University School of Dentistry, Mizuho, Japan.
[2]Research Center for Asian Infectious Diseases, The Institute of Medical Science, The University of Tokyo, Tokyo, Japan.
[3]Division of Cellular and Molecular Biology, The Institute of Medical Science, The University of Tokyo, Tokyo, Japan.
[4]Medical Genome Center, National Center for Geriatrics and Gerontology, Obu, Japan.

*Correspondence should be addressed to T.I. (into@dent.asahi-u.ac.jp)

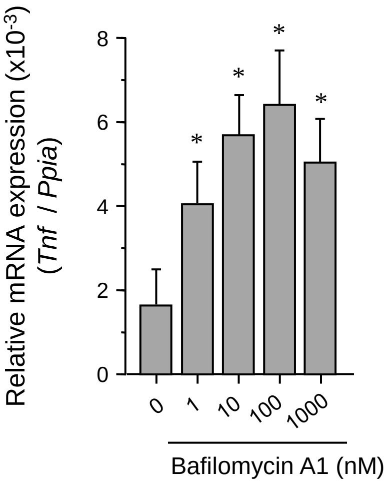

Supplementary Figure 1. Induction of mRNA expression of inflammatory cytokines by BafA1 treatment.
Mouse BMDMs were incubated with the indicated concentrations of BafA1 for 12 h, followed by RNA extraction. Relative expression levels of *Tnf* were determined by qRT-PCR. Each value is expressed as mean ± SD (n = 3); **p* < 0:05 (versus 0 nM), one-way ANOVA and Dunnett's test for post-hoc comparisons (μc<μi).

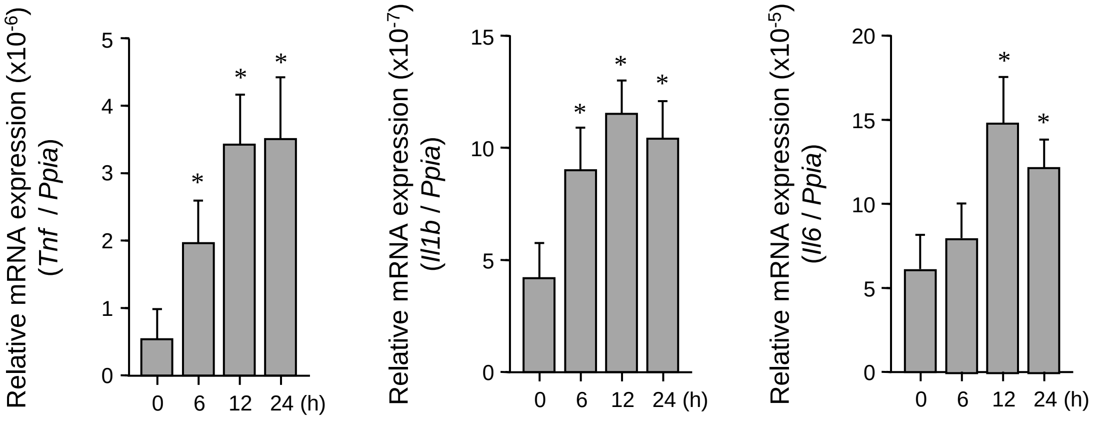

Supplementary Figure 2. Induction of mRNA of inflammatory cytokines by BafA1 treatment in MEFs.
MEFs were incubated with 100 nM BafA1 for the indicated periods, followed by RNA extraction. Relative expression levels of *Tnf*, *Il1b*, and *Il6* were determined by qRT-PCR. Each value is expressed as mean ± SD (n = 3); **p* < 0:05 (versus 0 h), one-way ANOVA and Dunnett's test for post-hoc comparisons (μc<μi).

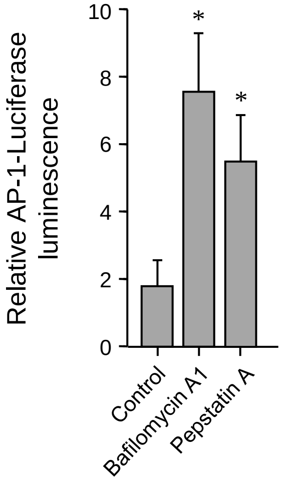

Supplementary Figure 3. Treatment with BafA1 or pepstatin A activates AP-1-dependent transcriptional activity.

An AP-1-driven luciferase reporter assay was performed on RAW264.7 macrophages. The cells were incubated with 0.5% DMSO (control), 100 nM BafA1, or 2 μM pepstatin A for 12 h. Relative AP-1 activity was measured, and the data are expressed as mean ± SD (n = 3); *p < 0:05 (versus Control), one-way ANOVA and Dunnett's test for post-hoc comparisons (μc<μi).

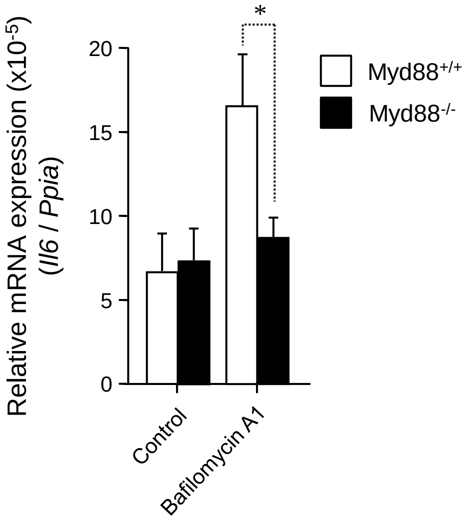

Supplementary Figure 4. Deficiency in MyD88 impairs BafA1-induced *Il6* expression in MEFs.

MEFs collected from Myd88$^{+/+}$ mice and Myd88$^{-/-}$ mice were incubated with 0.5% DMSO (control) or 100 nM BafA1 for 12 h, followed by RNA extraction. Relative expression levels of *Il6* were determined by qRT-PCR. Each value is expressed as mean ± SD (n = 3); *p < 0:05 (Myd88$^{+/+}$ versus Myd88$^{-/-}$), one-way ANOVA and Dunnett's test for post-hoc comparisons (μc≠μi).

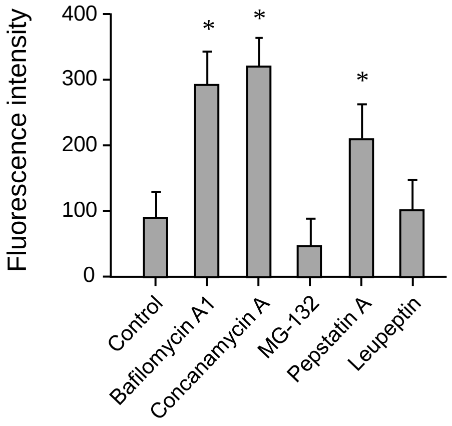

Supplementary Figure 5. Treatment with inhibitors of lysosomes or of lysosomal proteases increases MyD88 expression in MEFs.

MEFs cultured in lumox 24-well plates were incubated with 0.5% DMSO (control), 100 nM BafA1, 100 nM concanamycin A, 20 μM MG-132, 2 μM pepstatin A, or 50 μM leupeptin for 12 h, followed by immunofluorescent staining for MyD88. Quantification of MyD88 was performed by measurement of fluorescence intensity of the wells. Data are expressed as mean ± SD (n = 3); **p* < 0:05 (versus Control), one-way ANOVA and Dunnett's test for post-hoc comparisons (μc<μi).

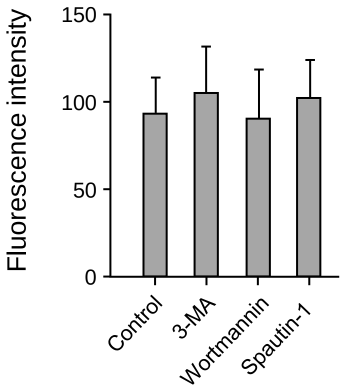

Supplementary Figure 6. Treatment with inhibitors of macroautophagy does not affect basal MyD88 expression.

MEFs cultured in lumox 24-well plates were treated with 0.5% DMSO (control), 10 mM 3-MA, 100 nM wortmannin, or 10 μM spautin-1 for 12 h, followed by immunofluorescent staining for MyD88. Quantification of MyD88 was performed by measurement of fluorescence intensity of the wells. Data are expressed as mean ± SD (n = 3)); **p* < 0:05 (versus Control), one-way ANOVA and Dunnett's test for post-hoc comparisons (μc<μi).

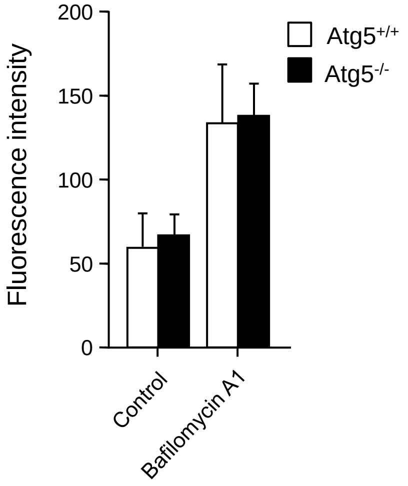

Supplementary Figure 7. Deficiency in ATG5 does not affect BafA1 treatment-stimulated basal MyD88 expression. $Atg5^{+/+}$ MEFs and $Atg5^{-/-}$ MEFs were incubated with 0.5% DMSO (control) or 100 nM BafA1 for 12 h, followed by immunofluorescent staining for MyD88. Quantification of MyD88 was performed by measurement of fluorescence intensity of the wells. Data are expressed as mean ± SD (n = 3); **p* < 0:05 ($Atg5^{+/+}$ versus $Atg5^{-/-}$), one-way ANOVA and Dunnett's test for post-hoc comparisons (μc≠μi).

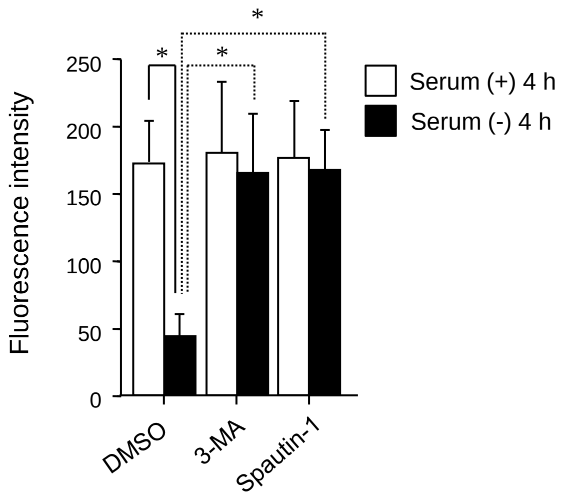

Supplementary Figure 8. Starvation-induced degradation of basal MyD88 is attenuated by inhibitors of macroautophagy. MEFs were incubated with or without 10 mM 3-MA or 10 μM spautin-1 for 6 h, and then cultured under the starvation conditions for 4 h, followed by immunofluorescent staining for MyD88. Quantification of MyD88 was performed by measurement of fluorescence intensity of the wells. Data are expressed as mean ± SD (n = 3); **p* < 0.05, two-way ANOVA and multiple Dunnett's tests for post-hoc comparisons between the groups of interest (μc≠μi).

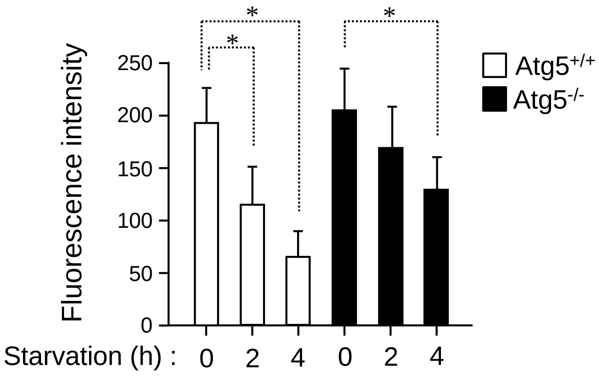

Supplementary Figure 9. Starvation-induced degradation of basal MyD88 is affected by ATG5 deficiency. Atg5$^{+/+}$ MEFs and Atg5$^{-/-}$ MEFs were cultured under the starvation conditions for the indicated periods, followed by immunofluorescent staining for MyD88. Quantification of MyD88 was performed by measurement of fluorescence intensity of the wells. Data are expressed as mean ± SD (n = 3); *$p < 0.05$, two-way ANOVA and multiple Dunnett's tests for post-hoc comparisons between the groups of interest (μc≠μi).

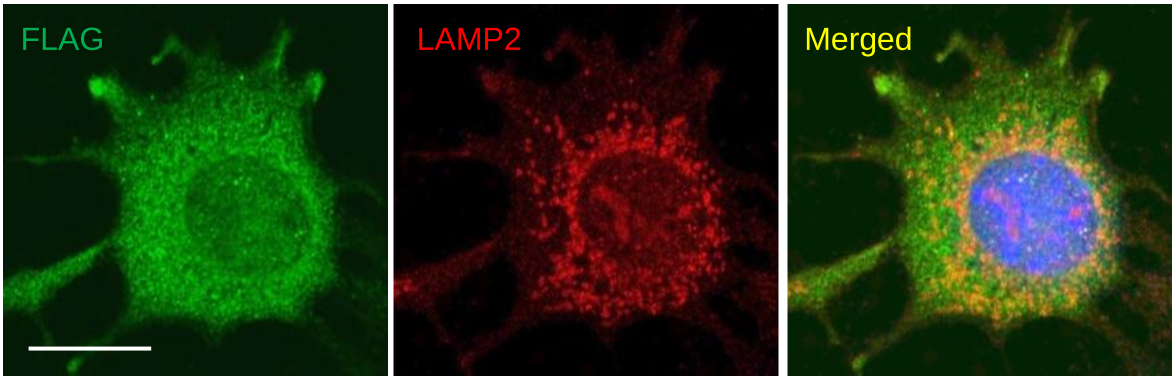

Supplementary Figure 10. MyD88-GyrB is expressed as speckles in Myd88$^{-/-}$ MEFs. Myd88$^{-/-}$ MEFs stably expressing FLAG-tagged MyD88-GyrB were prepared, and immunofluorescent staining for FLAG (green) and LAMP2 (red) was carried out. Cell nuclei were stained with Hoechst 33342. Images were acquired by means of a confocal microscope. Scale bar: 10 μm.

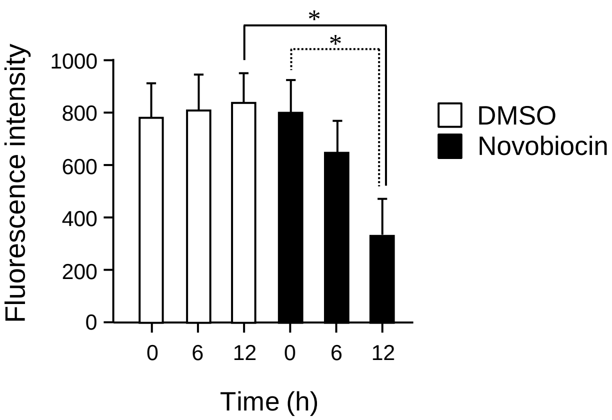

Supplementary Figure 11. Monomerization of MyD88-GyrB promotes its degradation.
Myd88$^{-/-}$ MEFs stably expressing FLAG-tagged MyD88-GyrB were incubated with 10 μM novobiocin for the indicated periods, followed by immunofluorescent staining for FLAG. Quantification of MyD88-GyrB was performed by measurement of fluorescence intensity of the wells. Data are expressed as mean ± SD (n = 3); *$p < 0.05$, two-way ANOVA and multiple Dunnett's tests for post-hoc comparisons between the groups of interest (μc≠μi).

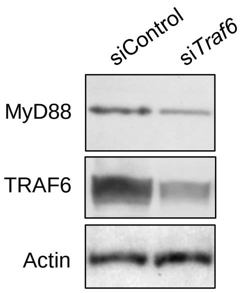

Supplementary Figure 12. Basal expression of MyD88 is lowered along with a decrease in TRAF6 expression. MEFs were transfected with siRNA against *Traf6* or with control siRNA. The cells were lysed, and the protein levels of MyD88, TRAF6, and actin were assessed by immunoblotting. All the blots were obtained under the same experimental conditions, and the cropped images of the blots are shown. The uncropped images are in Supplementary Fig. 26.

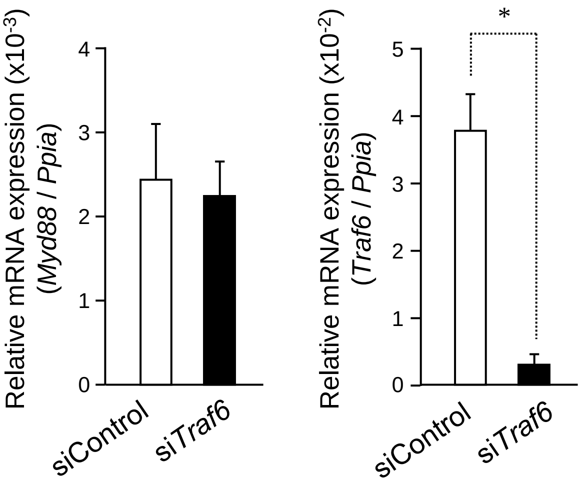

Supplementary Figure 13. A knockdown of *Traf6* does not affect *Myd88* transcription.
MEFs were transfected with siRNA against *Traf6* or with control siRNA, followed by RNA extraction. Relative expression levels of *Myd88* and *Traf6* were assessed by qRT-PCR. Each value is expressed as mean ± SD (n = 3); *p < 0.01, Student's t-test.

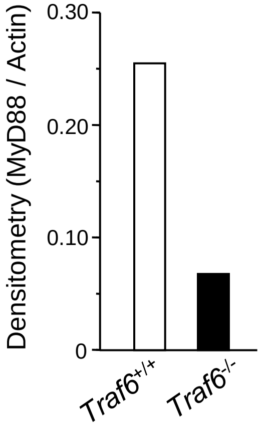

Supplementary Figure 14. Densitometric analysis of the images shown in Fig. 6a.

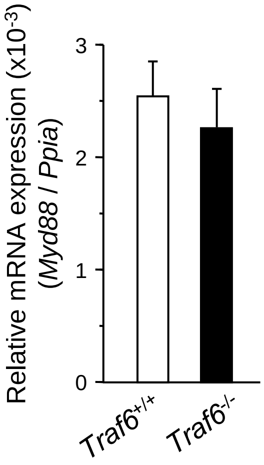

Supplementary Figure 15. Deficiency in *Traf6* does not affect *Myd88* transcription.
Traf6$^{+/+}$ MEFs and Traf6$^{-/-}$ MEFs were prepared, and total RNA was extracted. Relative expression levels of *Myd88* were assessed by qRT-PCR. Each value is expressed as mean $\pm$ SD (n = 3); *$p < 0.05$, Student's *t*-test.

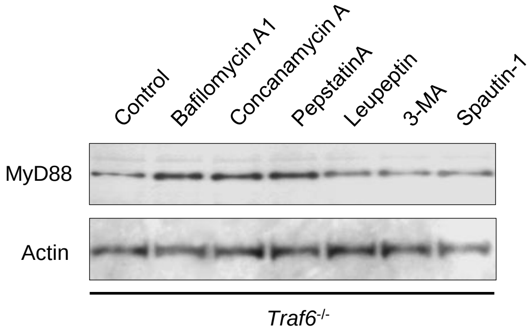

Supplementary Figure 16. Downregulation of basal MyD88 in Traf6$^{-/-}$ cells can be attenuated by lysosomal inhibitors.
Traf6$^{-/-}$ MEFs were incubated with 0.5% DMSO (control), 100 nM BafA1, 100 nM concanamycin A, 2 μM pepstatin A, 50 μM leupeptin, 10 mM 3-MA, or 10 μM spautin-1 for 12 h, followed by cell lysis. Protein levels of MyD88 and actin were assessed by immunoblotting. All the blots were obtained under the same experimental conditions, and the cropped images of the blots are shown.

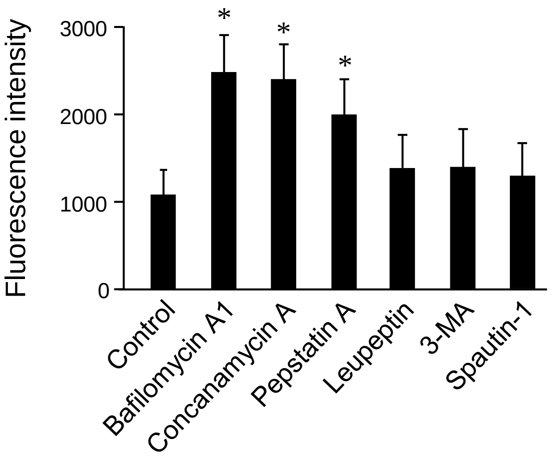

Supplementary Figure 17. Downregulation of MyD88-GyrB in Traf6$^{-/-}$ cells can be attenuated by lysosomal inhibitors.

Traf6$^{-/-}$ MEFs stably expressing FLAG-tagged MyD88-GyrB were treated with 0.5% DMSO (control), 100 nM BafA1, 100 nM concanamycin A, 2 μM pepstatin A, 50 μM leupeptin, 10 mM 3-MA, or 10 μM spautin-1 for 12 h, followed by immunofluorescent staining for FLAG. Quantification of MyD88-GyrB protein was performed by measurement of fluorescence intensity of the wells. Data are expressed as mean ± SD (n = 3); *p < 0:05 (versus Control), one-way ANOVA and Dunnett's test for post-hoc comparisons (μc<μi).

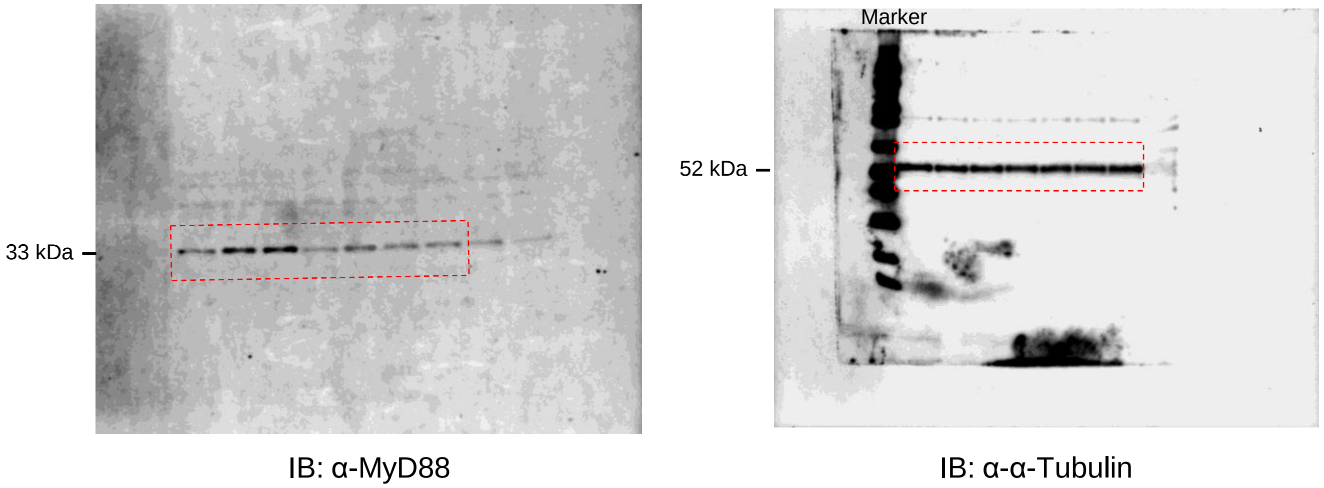

Supplementary Figure 18. Uncropped images of immunoblot results of Fig. 2b.
The full-length blots, showing MyD88 and α-tubulin, were obtained under the same experimental conditions. The cropped parts indicated by the boxes with red dashed lines are shown in Fig. 2b.

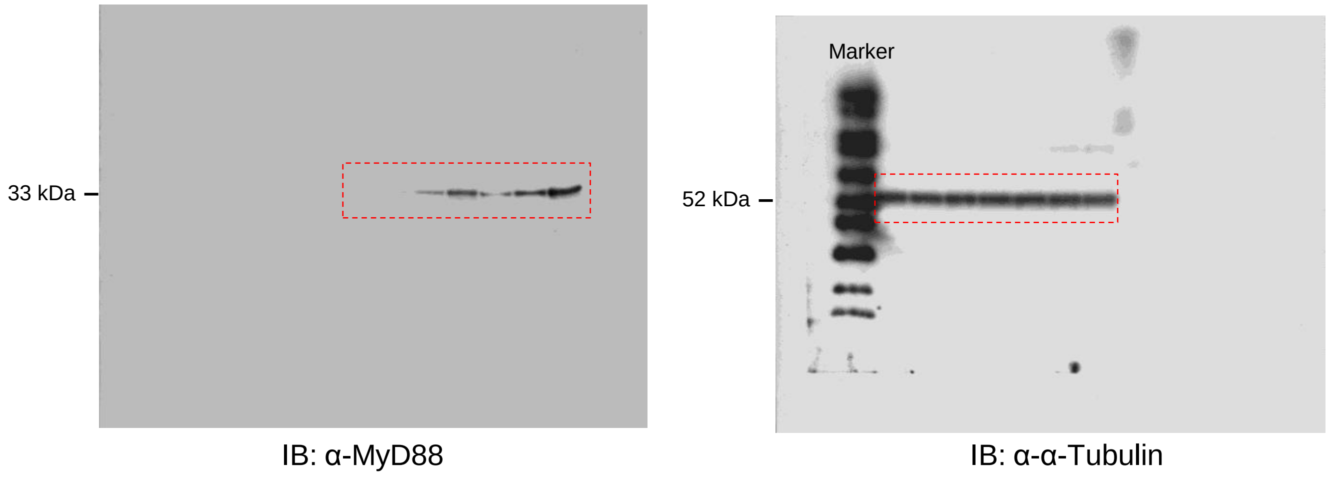

Supplementary Figure 19. Uncropped images of immunoblot results of Fig. 3b.
The full-length blots, showing FLAG-tagged MyD88 and α-tubulin, were obtained under the same experimental conditions. The cropped parts indicated by the boxes with red dashed lines are shown in Fig. 3b.

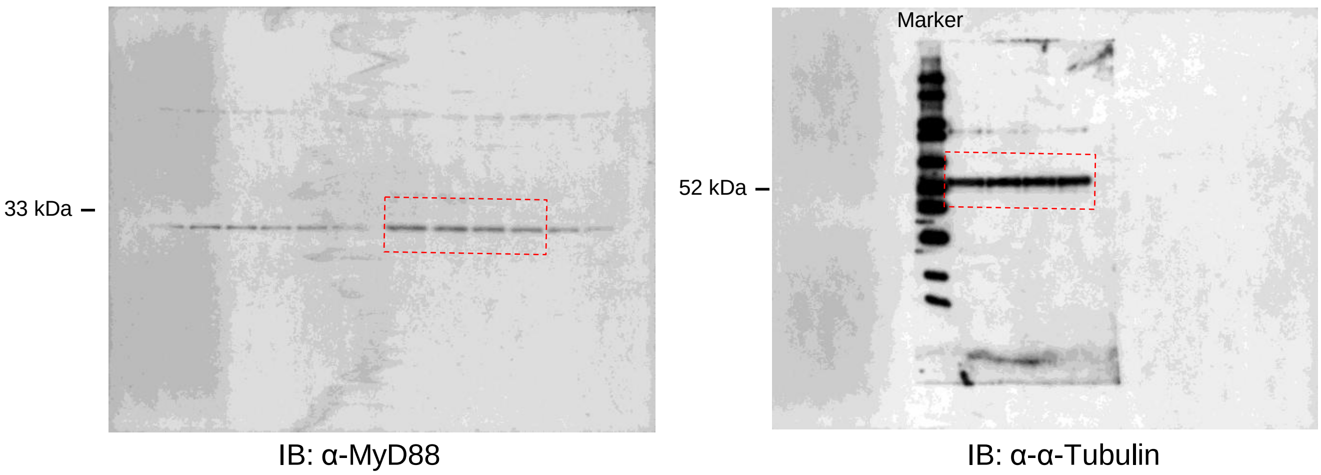

Supplementary Figure 20. Uncropped images of immunoblot results of Fig. 4a.
The full-length blots, showing MyD88 and α-tubulin, were obtained under the same experimental conditions. The cropped parts indicated by the boxes with red dashed lines are shown in Fig. 4a.

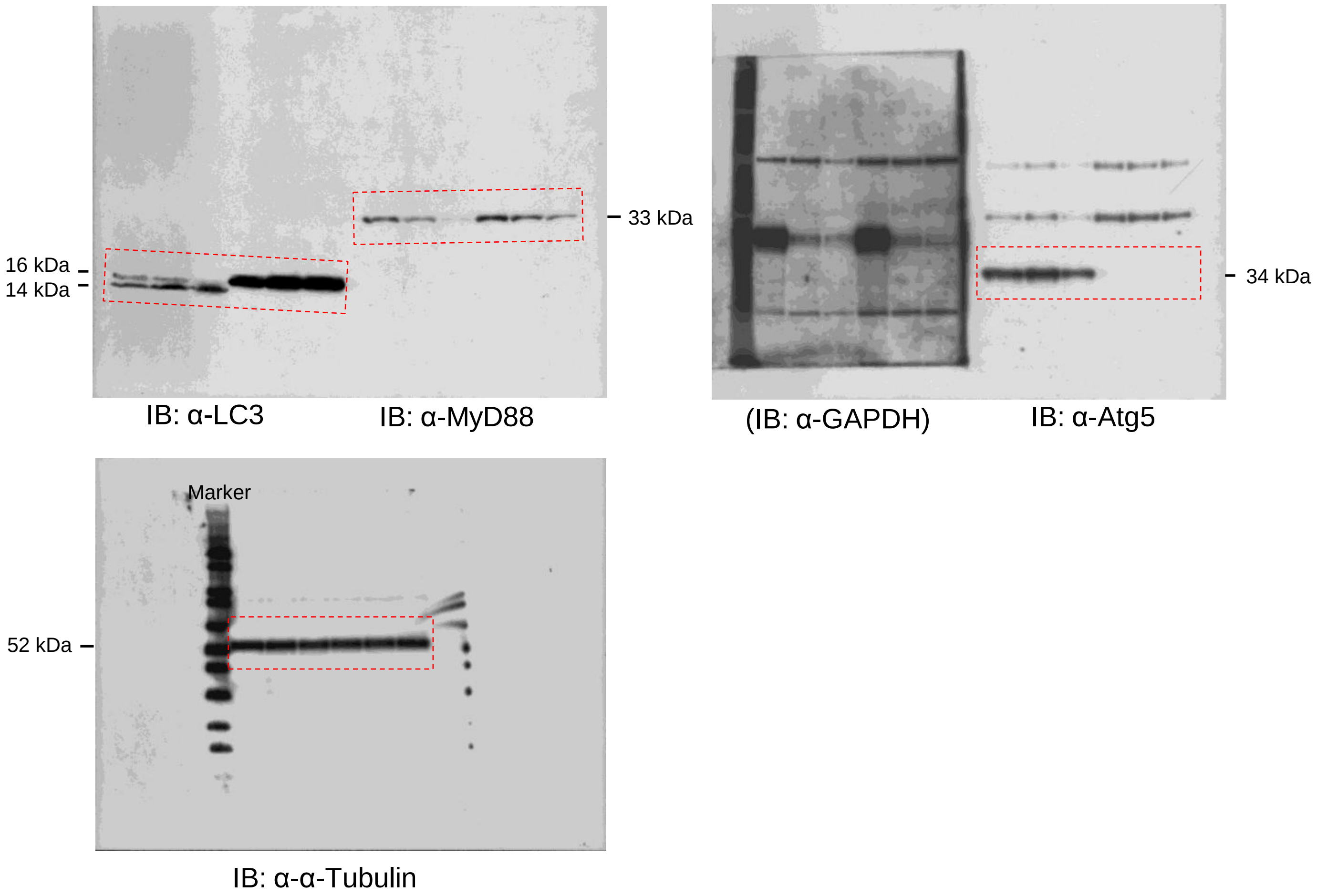

Supplementary Figure 21. Uncropped images of immunoblot results of Fig. 4d.
The full-length blots of MyD88, LC3, Atg5 and α-tubulin, were obtained under the same experimental conditions. The cropped parts indicated by the boxes with red dashed lines are shown in Fig. 4d.

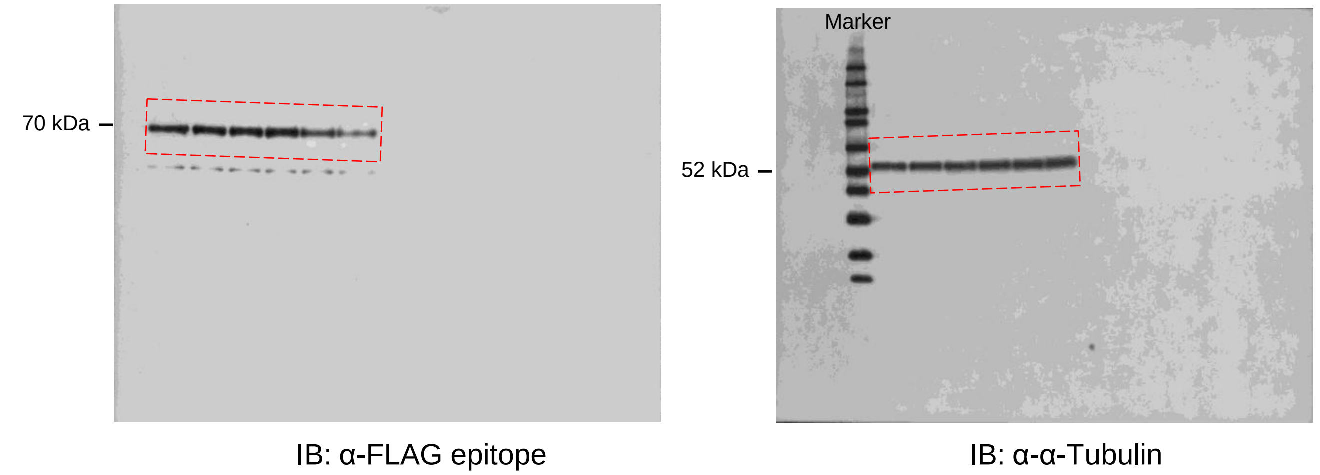

Supplementary Figure 22. Uncropped images of immunoblot results of Fig. 5a.
The full-length blots, showing FLAG-tagged MyD88-GyrB and α-tubulin, were obtained under the same experimental conditions. The cropped parts indicated by the boxes with red dashed lines are shown in Fig. 5a.

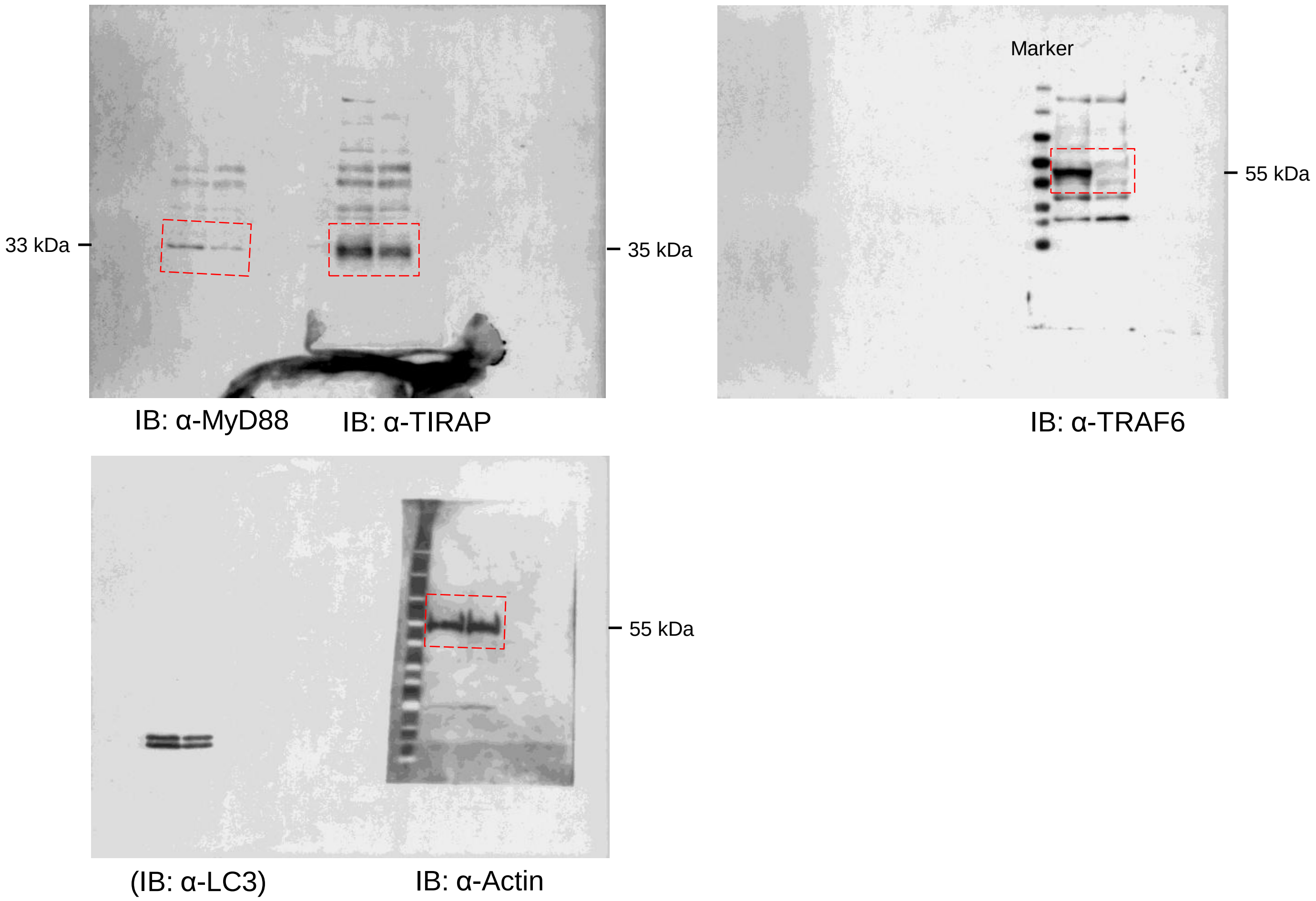

Supplementary Figure 23. Uncropped images of immunoblot results of Fig. 6a.
The full-length blots of MyD88, TIRAP, TRAF6 and actin, were obtained under the same experimental conditions. The cropped parts indicated by the boxes with red dashed lines are shown in Fig. 6a.

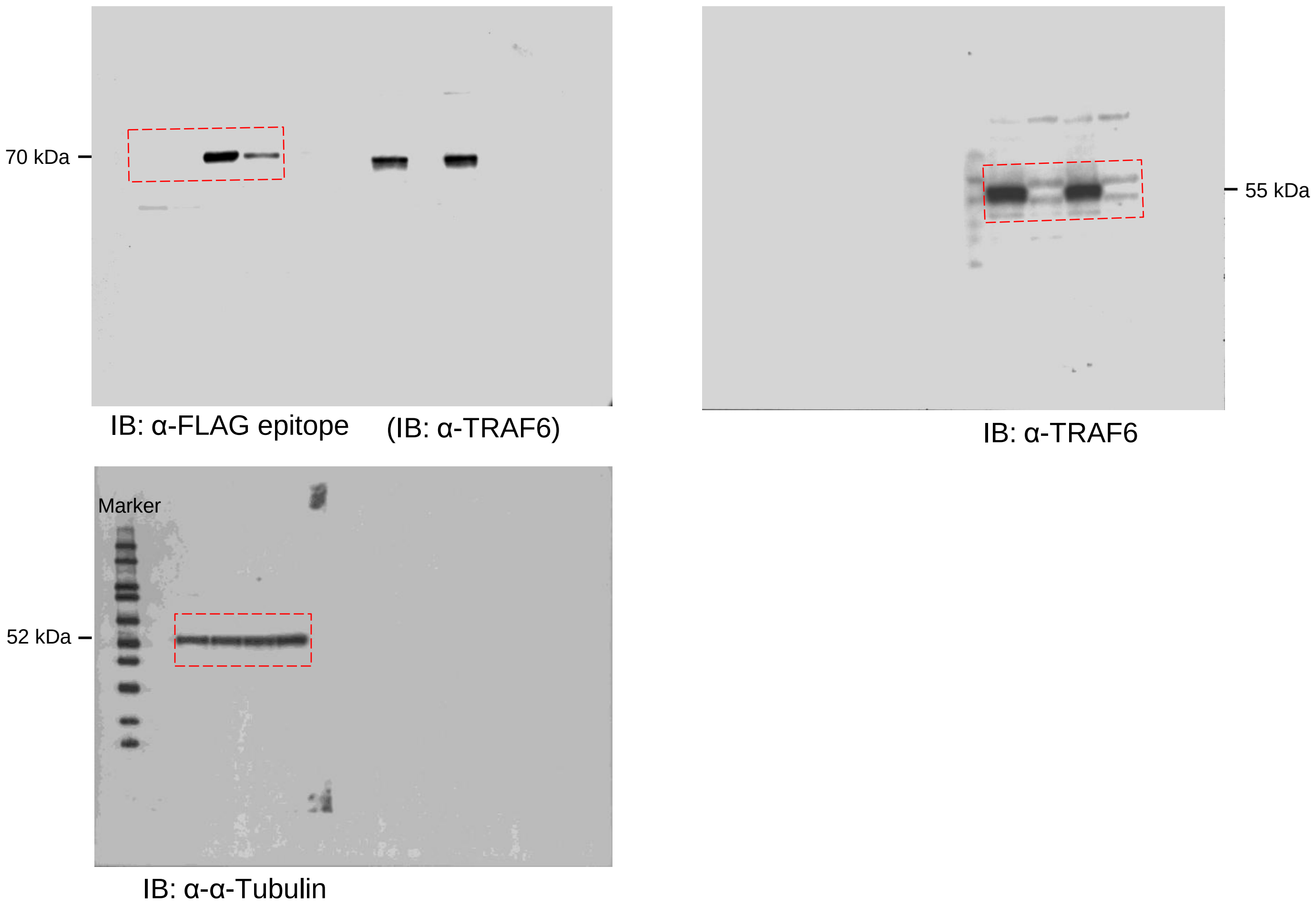

Supplementary Figure 24. Uncropped images of immunoblot results of Fig. 6c.
The full-length blots of FLAG-tagged MyD88-GyrB, TRAF6 and α-tubulin, were obtained under the same experimental conditions. The cropped parts indicated by the boxes with red dashed lines are shown in Fig. 6c.

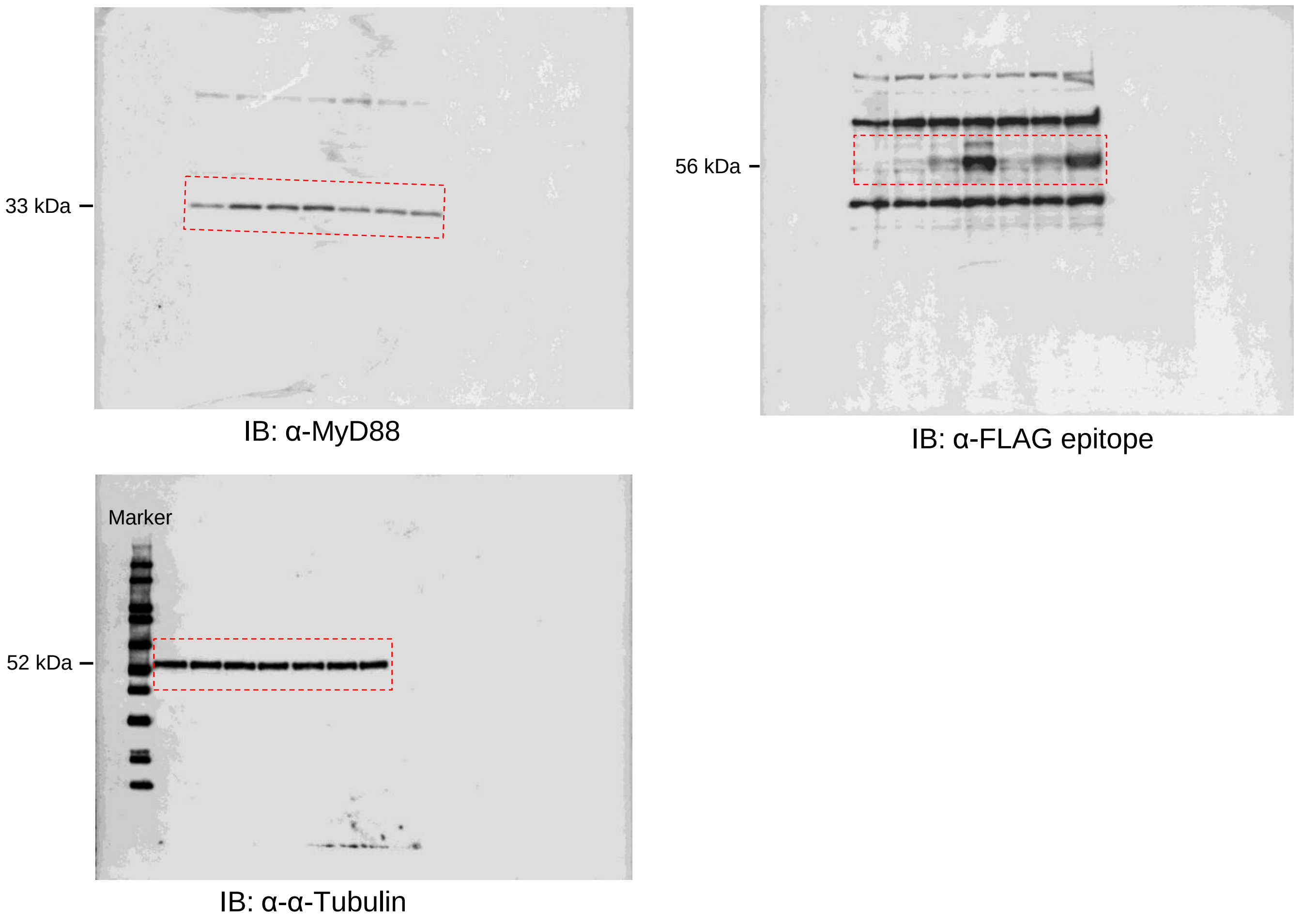

Supplementary Figure 25. Uncropped images of immunoblot results of Fig. 6e.
The full-length blots of MyD88, FLAG-tagged TRAF6 and α-tubulin, were obtained under the same experimental conditions. The cropped parts indicated by the boxes with red dashed lines are shown in Fig. 6e.

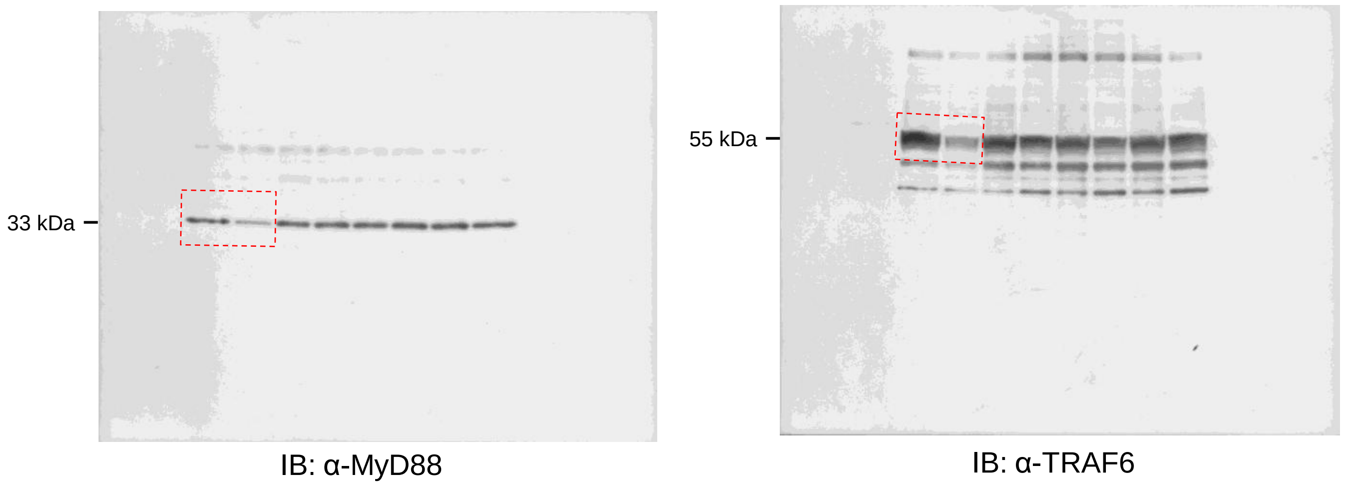

Supplementary Figure 26. Uncropped images of immunoblot results of Supplementary Fig. 12.
The full-length blots of MyD88 and TRAF6, were obtained under the same experimental conditions. The cropped parts indicated by the boxes with red dashed lines are shown in Supplementary Fig. 12.
